# Genetic overlap between sarcoidosis and lung cancer: a combined in silico and in vitro approach

**DOI:** 10.1186/s41065-025-00503-7

**Published:** 2025-08-12

**Authors:** Sanjukta Dasgupta, Moupiya Ghosh, Subhendu Chakrabarty, Gopal Chakrabarti, Amlan Das

**Affiliations:** 1 Department of Biotechnology, Center for Multidisciplinary Research & Innovations, Brainware University, 398, Ramkrishnapur Rd, near Jagadighata Market, Barasat, Kolkat, West Bengal 700125 India; 2 Department of Basic Science and Humanities, Institute of Engineering and Management (IEM), Kolkata, University of Engineering and Management, Newtown, Kolkata, West Bengal 700160 India; 3 Department of Biotechnology and Dr. B.C. Guha Centre for Genetic Engineering and Biotechnology, University of Calcutta, Kolkata, West Bengal 700019 India; 4 Department of Microbiology, M.U.C Women′s College, Burdwan, West Bengal 713104 India; 5 Department of Microbiology, Royal School of Biosciences, The Assam Royal Global University, Assam, 781035 India

**Keywords:** Sarcoidosis, Lung cancer, Metformin, Differentially expressed genes, X-ray diffraction

## Abstract

Sarcoidosis patients exhibit an elevated risk of developing lung cancer (LC), suggesting shared genetic and molecular mechanisms between these conditions. This study aimed to identify common differentially expressed genes (DEGs) in sarcoidosis and LC and to evaluate the therapeutic potential of a repurposable drug targeting these shared genes. Gene expression datasets (GSE157671 and GSE229253) were analyzed to identify overlapping DEGs, with validation performed using additional GEO datasets and the GEPIA tool. Functional enrichment and protein–protein interaction (PPI) analyses were conducted using Enrichr and STRING, while associated miRNAs and transcription factors were identified via miRNet. Twelve DEGs—*SALL4*, *WNT10A*, *RASAL1*, *CAMK2B*, *GADD45B*, *KLF4*, *OLR1*, *CSF3*, *WIF1*, *RAMP3*, *AGER*, and *PRKAG3*—were consistently dysregulated in both diseases. These genes were significantly associated with epithelial cell enrichment and the Wnt signaling pathway. Drug–gene interaction analysis using DGIdb prioritized metformin as a candidate drug targeting PRKAG3. Its structural integrity was confirmed via X-ray diffraction (XRD) and Rietveld refinement. In vitro validation using MTT assays revealed that metformin selectively reduced viability in A549 (adenocarcinoma human alveolar basal epithelial cells) and HeLa (a widely used epithelial cancer cell line), with minimal cytotoxicity in WI38 normal lung fibroblasts. Colony formation assays further demonstrated dose-dependent, long-term growth inhibition in cancer cells, corroborated by observable morphological alterations. Overall, this study highlights shared pathogenic signatures between sarcoidosis and LC and proposes metformin as a promising therapeutic candidate. These findings support the rationale for drug repurposing and the development of targeted therapies for patients with overlapping disease profiles or those at increased risk of LC progression from sarcoidosis.

**Supplementary Information:**

The online version contains supplementary material available at 10.1186/s41065-025-00503-7.

## Introduction

Sarcoidosis is a multisystem inflammatory disease characterized by the formation of non-caseating granulomas in various organs, most commonly the lungs and lymph nodes. The etiology of sarcoidosis remains unclear, but it is believed to result from an exaggerated immune response to an unknown antigen. Clinical presentation varies widely, ranging from asymptomatic cases to severe, debilitating illnesses. The symptoms often include persistent dry cough, fatigue, weight loss, and dyspnea. Diagnosis of sarcoidosis is typically confirmed through clinical evaluation, radiological imaging studies (chest X-rays or high-resolution computed tomography), and histological examination of tissue biopsies showing granulomas without evidence of infection or malignancy [1]. Epidemiological studies have suggested that individuals with sarcoidosis have an increased risk of developing lung cancer (LC). This could be attributed to chronic inflammation and fibrosis caused by persistent granulomatous inflammation, potentially leading to malignant transformation [2]. Distinguishing between sarcoidosis-related nodules and primary LC can be often challenging, as both conditions exhibit similar radiological features, necessitating a thorough and careful differential diagnosis.

Several studies have reported a higher incidence of LC among patients with pulmonary sarcoidosis. For example, Bonifazi et al. [3] demonstrated a statistically significant increase in LC risk among sarcoidosis patients, suggesting a potential causal link between the two conditions. Similarly, a recent comprehensive literature review by reinforced the association between sarcoidosis and subsequent LC development [4]. However, findings remain inconsistent, notably, a population-based study found no significant association between the two diseases, underscoring the need for further large-scale, mechanistic studies to elucidate this relationship [5]. Overall, while growing evidence suggests an increased risk of LC in sarcoidosis patients, the underlying genetic mechanisms remain poorly understood. There is a clear knowledge gap regarding the shared genetic and molecular drivers that may contribute to both diseases. Immunosuppressive therapies commonly used in sarcoidosis, such as corticosteroids, may elevate cancer risk by impairing immune surveillance. Therefore, identifying not only the common genetic features but also therapeutic agents capable of targeting these shared molecules is crucial for advancing risk stratification and treatment strategies for patients affected by both conditions.

Recent advances in bioinformatics-based analyses have greatly improved our understanding of dysregulated genes, offering critical insights into the genetic underpinnings of complex pulmonary diseases [6–8]. Integrating large-scale transcriptomic datasets, such as those from the Gene Expression Omnibus (GEO), facilitates the identification of key molecular pathways driving the complex diseases, enabling targeted therapeutic strategies [9]. Advancements in computational biology have significantly accelerated drug discovery, particularly through bioinformatics-driven identification of potential therapeutic compounds that target overlapping molecular signatures between related conditions, such as sarcoidosis and LC. While, in silico approaches streamline the initial phases of drug discovery, experimental validation is essential to confirm efficacy and safety. X-ray diffraction (XRD) analysis, particularly when combined with Rietveld refinement, provides precise structural insights into drug candidates, ensuring their suitability for further development. Furthermore, evaluating drug effects across a range of cell lines—such as A549 (adenocarcinoma human alveolar basal epithelial cells), HeLa (a widely used epithelial cancer cell line), and WI38 (normal lung fibroblasts)—provides critical insights into the drug’s selective cytotoxicity toward cancer versus normal cells. Such multi-layered validation bridges the gap between computational predictions and clinical applicability, reinforcing assurance in drug repurposing strategies for complex conditions like sarcoidosis-associated LC.

This study aims to identify dysregulated genes common to sarcoidosis and LC, investigate their functional and clinical relevance, and explore metformin as a potential therapeutic agent. By integrating bioinformatics, structural validation, and in vitro analysis, this report provides a comprehensive approach toward understanding shared molecular pathways and potential therapeutic interventions.

## Materials and methods

### Identification of the common differentially expressed genes (DEGs)

**Fig. 1:**
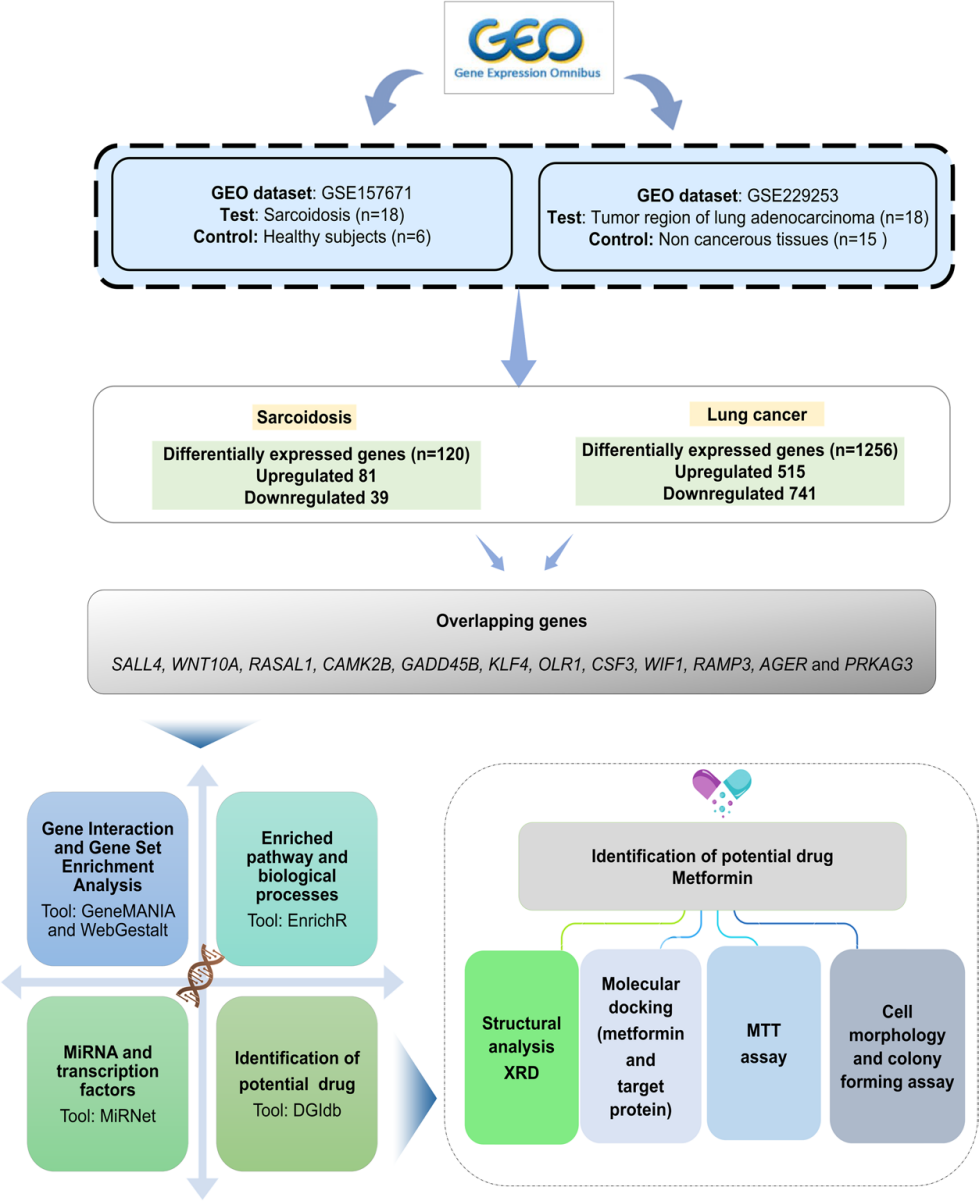
Workflow of the study design

The data analyzed in this study were obtained from the publicly available NCBI-GEO database (http://www.ncbi.nlm.nih.gov/geo/). The keywords ‘‘sarcoidosis’’ and ‘‘lung cancer” were used to find relevant datasets (last accessed: May 5, 2024). The focus was specifically on studies that provided comprehensive gene expression data related to both sarcoidosis and LC. Since majority of the studies suggest a relationship between lung adenocarcinoma and granulomatous lung diseases [10–12], we selected lung adenocarcinoma as the representative subtype within the LC group to enable a more meaningful comparison with sarcoidosis. The overall study design is illustrated in Fig. 1.

GSE157671 and GSE229253, which highlight altered gene expression profiles in sarcoidosis and lung adenocarcinoma, were selected for inclusion in this study. Ethics committee approval was not required because the study is based on open-source data. GSE157671 dataset includes micro-dissected lung granulomas from eighteen sarcoidosis, four tuberculosis, three coccidioidomycosis, and six healthy controls. Since the present study is focused on patients with sarcoidosis, only gene expression data from sarcoidosis patients and healthy controls were included in the analysis. The majority of sarcoidosis subjects were female and non-Hispanic whites. The baseline characteristics of the recruited subjects are shown in Table 1 [13, 14]. The GSE229253 dataset contains thirty-three lung tissue samples, including eighteen samples from the tumor region of lung adenocarcinoma and fifteen from tumor-adjacent lung. The data was analyzed using a high-throughput sequencing Illumina NovaSeq 6000 platform [15].

**Table 1:** Baseline characteristics of the recruited subjects (GSE157671)

Variable	Sarcoidosis	Coccidiodomycosis	Tuberculosis
Number of cases	18	3	4
Granuloma origin			
Lymph node (mediastinal)	12	0	2
Lung	6	3	2
Sex, female, N (%)	66	33	50
Age, (mean, Sd)	52.8 ± 11.4	38.3 ± 16.5	58.3 ± 9.3
Race/ethnicity, N (%)			
Black or African American	11	0	0
White	62	100	100
Hispanic*	17	66	75
Other**	11	na	na

There is no significant age difference (*p* = 0.1). The majority of participants across all three granulomatous diseases were White. Among the sarcoidosis patients, 11% identified as Black or African American, while half of the Hispanic individuals also identified as White. **The"Other"category included Native American and Multiracial individuals

### Screening of common DEGs

Data analysis was conducted using the GEO2R package. GEO2R utilizes GEOquery and limma for comparative gene expression analysis [16]. DEGs were selected based on the criteria of |log₂(fold change)|> 1.75, an adjusted *p*-value < 0.005, and a raw *p*-value < 0.0001 for both sarcoidosis and LC patients compared to their respective healthy controls. The common DEGs identified from both comparisons were used as the primary dataset in this study to explore shared molecular mechanisms and potential therapeutic targets.

### Validation of altered DEGs

To further corroborate the findings, the expression of the common DEGs identified in the discovery datasets of sarcoidosis and LC was validated using independent datasets. In the case of sarcoidosis, three additional NCBI-GEO datasets were utilized for this purpose: GSE19314 [17, 18], GSE162712 [19], and GSE192829 [20]. These datasets were carefully selected based on their relevance and robust sample size to ensure comprehensive validation across different cohorts. For LC, the expression patterns of the same set of common genes were validated using two well-established datasets, GSE18842 [21] and GSE103888 [22], which encompass diverse clinical samples of LC patients. To further reinforce the reliability of the gene expression findings, the GEPIA (Gene Expression Profiling Interactive Analysis) tool was employed. This tool integrates TCGA and GTEx datasets, enabling cross-validation of gene expression in a large-scale setting and offering insights into differential expression patterns across various cancer types, including LC [23]. This multi-layered validation approach reinforces the reliability of the discovery-phase results by confirming the differential expression of the identified genes in both sarcoidosis and LC.

### Survival analysis and TNM stage correlation in LC based on common DEGs

Kaplan–Meier Plotter (http://www.kmplot.com), which evaluates the prognostic relevance of 54,675 genes using data from 10,461 samples—including approximately 2,437 LC patients, was used in this study to assess the survival patterns associated with the query common genes. Survival information is sourced from major public databases, including GEO, EGA, and TCGA [24]. This tool applies Cox regression analysis to calculate overall hazard ratios (HRs) and determine the survival impact of each identified DEGs in LC.

To further explore the relationship between gene expression and tumor progression, GEPIA violin plots based on TCGA clinical annotations were used. It assesses the association between gene expression levels and tumor-node-metastasis (TNM) stages of LC. ANOVA was performed to evaluate statistical significance across different cancer stages, with corresponding F-values and *p*-values reported for each gene.

### Gene and protein–protein interaction network

GeneMANIA (http://www.genemania.org), a user-friendly web interface, was employed to construct the gene network for the common genes between sarcoidosis and LC. This tool enables the analysis of co-expression and colocalization among the identified genes [25]. Additionally, the protein–protein interaction (PPI) network, which underscores the intricate web of interactions between the proteins, was constructed using the STRING web tool. This tool integrates diverse biological data sources to predict functional associations among proteins [26]. This network provides valuable insights into the complex interactions between proteins, offering a comprehensive perspective on their functional relationships.

### Gene-miRNA interaction network and pathway analysis

MiRNet tool, an integrated platform designed to correlate the miRNAs with genes, was used to develop the miRNA–DEG interaction network. Interaction data from MiRNet were derived from miRTarBase v7.0, TarBase v7.0, and miRecords, which compile gene–miRNA associations. The most connected miRNAs with the common genes between sarcoidosis and LC were identified by analysing degree and betweenness centralities [27].

Enrichr (https://maayanlab.cloud/Enrichr/) was also employed to investigate the pathways associated with the common genes, identifying the most significantly enriched overlapping pathway between sarcoidosis and LC [28].

### Enriched immune cells

Enriched immune cells associated with these DEGs were further identified using the Web-based Cell-type Specific Enrichment Analysis of Genes (WebCSEA) tool [29]. This platform utilizes comprehensive gene expression datasets to identify specific immune cell types that are significantly enriched with the input query genes (common DEG between sarcoidosis and LC). This analysis provides an insight into the immune landscape associated with the identified DEGs, offering evidence of the immunological mechanisms potentially underlying sarcoidosis and LC pathogenesis.

### Prediction of structure of promising drug

To identify potential drugs that target common DEGs between sarcoidosis and LC, Drug Gene Interaction Database (DGIdb) was utilized. DGIdb is a comprehensive resource that includes over 14,144 drug-gene interactions, encompassing 2,611 human genes and 6,307 drugs. By entering the names of the commonly altered genes into the DGIdb interface, a list of potential drugs that interact with these specific genes can be generated [30]. This approach is crucial for identifying drugs that may modulate the activity of shared genes and thus possess therapeutic potential [31].

### Structural and microstructural characterization of the drug by XRD

The structural properties of the selected drug, metformin, were characterized using X-ray powder diffraction (XRD). XRD patterns were obtained with a Bruker D8 Advance diffractometer (da Vinci model) operating at 40 kV and 40 mA, utilizing Ni-filtered CuKα radiation (λ = 1.5418 Å). Using a step size of 0.02˚ 2θ and a scan time of 5 s per step, the XRD pattern was captured in step scan mode within the scattering angle of 20˚−80˚2θ.

### Rietveld refinement of XRD pattern

It is well established that analysis of the XRD pattern enables the identification of various structural and microstructural properties of a candidate drug, including crystallinity, phase composition, lattice parameters, and crystallite size. There are many reflections in the XRD pattern. The Rietveld refinement technique is an effective way to determine the lattice parameters, crystallite size, r.m.s. lattice strain, and other structural and microstructural properties of the crystalline phase that makes up the drug.

Based on the structural data that was provided for the separately recognized phase that was present in the metformin from their corresponding ICSD/COD files, an XRD pattern (Ic) was simulated using the Rietveld software MAUD 2.7. After refining a number of phase structural and microstructural parameters as well as certain provided instrumental parameters, the simulated XRD pattern was then matched with the acquired experimental pattern (Io) [32, 33]. The degree 5 polynomial was employed to fit the background intensity. The"goodness of fit"(GoF), which is the ratio of expected residual error (Rexp) to weighted residual error (Rwp) and can be stated as GoF = Rexp/Rwp, was used to track the fitting process as it approached unity. An excellent fitting quality is indicated by a GoF value that is near to unity. The GoF values for metformin in the current investigation is 1.26, indicating a good fit for the XRD pattern.

### Cell viability assay

The effect of metformin on the viability of the A549 cell line (derived from adenocarcinoma human alveolar basal epithelial cells), HeLa cells (a well-known epithelial cancer cell line), and human normal lung fibroblast WI38 cells was tested using the MTT (3-[4,5-dimethylthiazol-2-yl]−2,5-diphenyltetrazolium bromide) assay. Cells were cultured in 96-well culture plate (10^4^ cells per well) in DMEM medium supplied with 5% CO2 under humidified conditions. Then cells were treated with different concentrations of metformin (0–400 µg/ml) for 48 h. MTT (5 mg/mL) was dissolved in PBS, added to each well (20 µL), and incubated until purple precipitate was visible. The precipitate was dissolved in 100 μL of Triton-X and absorbance was measured on an ELISA reader (MultiskanEX, Lab systems, Helsinki, Finland) at a test wavelength of 570 nm and a reference wavelength of 650 nm.

Cultured A549, HeLa, and WI38 cells were treated with metformin (0, 200, and 400 µg/ml) for 48 h, and morphological changes were observed using an Olympus CKX41 inverted microscope. Cell rounding and detachment were noted in A549 and HeLa cells at higher doses, while WI38 cells exhibited greater resistance.

### Colony formation assay

Cultured A549 and HeLa cells (1 × 10^4^ cells/ml initially seeded) were treated with varying concentrations of metformin (0–400 µg/ml) for 48 h. After replacing the media, cells from each set were trypsinized into single-cell suspension, and an equal number of cells (1 × 10^3^cells/ml) were seeded in 35 mm-Petri plates. The cells were then allowed further to grow for seven days. The viable colonies formed after incubation were stained with crystal violet. After staining and capturing image of the plates, the stain was dissolved in 10% acetic acid, and absorbance was taken at 600 nm using Multiskan EX plate reader, Lab systems, Helsinki, Finland.

## Results

### Identification of DEGs in sarcoidosis and LC compared to controls

Following the analysis of raw data, it was found that sarcoidosis patients exhibited 81 upregulated and 39 downregulated genes compared to the controls. In LC patients, 515 genes were elevated and 741 were decreased relative to controls. A Variable Importance in Projection (VIP) plot was generated for the altered genes to evaluate their contribution to the overall variance in the dataset. VIP scores reflect the relative importance of each feature, with higher values indicating greater relevance in distinguishing between disease and control groups [34, 35]. Similarly, mean–variance and mean dispersion plots were generated to visualize the distribution and variability of gene expression, ensuring that only high-confidence DEGs were selected based on the defined cut-off criteria. VIP, mean–variance, and mean dispersion plot of the altered genes in both sarcoidosis and LC as compared to their respective controls are shown in Supplementary Fig. 1.

### Common DEGs between sarcoidosis and LC

A comparative analysis of 120 DEGs identified in sarcoidosis and 1,256 DEGs in LC revealed twelve genes (*SALL4*, *WNT10A*, *RASAL1*, *CAMK2B*, *GADD45B*, *KLF4*, *OLR1*, *CSF3*, *WIF1*, *RAMP3*, *AGER*, and *PRKAG3*) that were commonly altered in both diseases compared to their respective controls. Supplementary Tables 1 and 2 provide details on the common significantly altered genetic signatures in both diseases. The fold change and corresponding false discovery rate (FDR) values for each DEG are presented in Supplementary Table 1 and 2.

Among the twelve common DEGs, *SALL4, WNT10A, RASAL1, CAMK2B* were significantly upregulated while *GADD45B, KLF4, OLR1, CSF3, WIF1, RAMP3* and *AGER* downregulated in both sarcoidosis and LC as compared to the controls*.* Additionally, *PRKAG3* was notably upregulated in sarcoidosis but decreased in LC patients. The common DEGs comprise 0.87% of the total 1376 DEGs.

### Validation of overlapping genes

The expression of the twelve overlapping genes was validated in both sarcoidosis and LC datasets to ascertain their consistency and relevance across both conditions, ensuring that the identified genes represent robust molecular signatures shared between the two diseases. The sarcoidosis datasets (GSE19314, GSE162712, and GSE192829) and LC datasets (GSE18842 and GSE103888) were analysed in comparison with their respective controls to validate the expression patterns of the twelve overlapping genes with GEO2R tool. To further support our findings, we reviewed multiple previously published studies that reported similar expression trends. Notably, studies by Sun et al. [36], Gatenby et al. [37], and Miao et al. [38] corroborated our results, reinforcing the involvement of these genes in inflammatory and oncogenic processes.

**Fig. 2:**
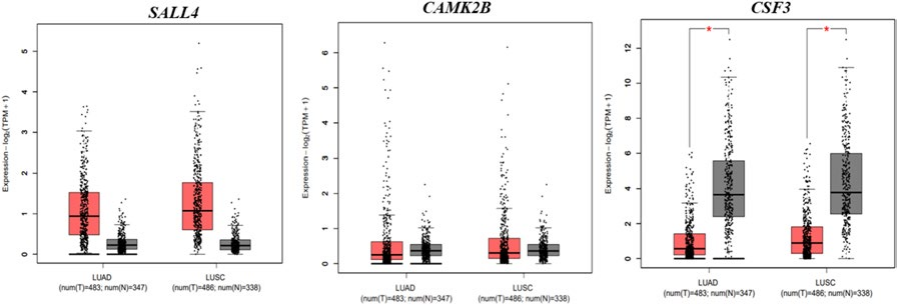
GEPIA box plots show the expression patterns of top three significantly dysregulated genes (*SALL4, CAMK2B, CSF3*) common between sarcoidosis and lung cancer

Additionally, to independently validate the altered gene expression with a specific focus on LC, we utilized the GEPIA online platform, which integrates transcriptomic data from TCGA and GTEx. This tool enabled us to further confirm the differential expression patterns of the candidate genes in LC samples compared to normal lung tissue. This tool could not be used for sarcoidosis, as it is specifically designed for analyzing gene expression in cancer and normal tissues, with no available datasets for sarcoidosis. The GEPIA box plots demonstrated a similar expression trend for the overlapping genes, with the expression of the top three DEGs (*SALL4, CAMK2B, CSF3*), based on log_2_ (fold change), presented in Fig. 2. Among the top three significantly dysregulated genes, *CSF3* exhibited significant dysregulation in tumor samples from both lung adenocarcinomas and lung squamous cell carcinomas cases compared to controls, with statistical significance (*p* < 0.05, independent t-test). However, no significant difference in expression was observed for *SALL4* and *CAMK2B* between tumor and control groups. The expression profiles of the remaining nine genes are provided in Supplementary Fig. 2.

### Overall stage wise association and survival pattern for LC

**Fig. 3:**
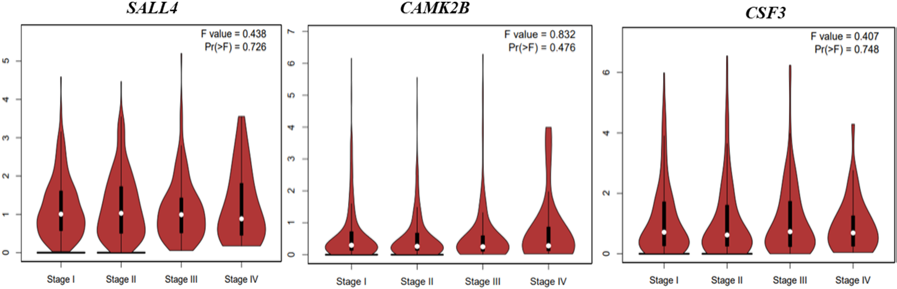
GEPIA violin plots showing the expression patterns of *SALL4*, *CAMK2B*, and *CSF3* across different tumor-node-metastasis (TNM) stages in lung cancer. ANOVA analysis resulted in F-values of 0.43, 0.40, and 0.83, with corresponding Pr(> F) values of 0.72, 0.74, and 0.47, indicating no statistically significant differences between stages. While these genes exhibit dysregulation in sarcoidosis and lung cancer, their expression does not significantly vary across TNM stages

GEPIA violin plots, based on TCGA clinical annotations, indicated the association between gene expression levels and TNM stages, with Fig. 3 illustrating the expression patterns of the top three altered genes in LC patients. The violin showing the expression patterns of *SALL4*, *CAMK2B*, and *CSF3* across different TNM stages in LC. ANOVA analysis resulted in F-values of 0.43, 0.40, and 0.83, with corresponding Pr(> F) values of 0.72, 0.74, and 0.47, indicating no statistically significant differences between stages. Supplementary Fig. 3 displaying the expression of the other nine genes in LC.

**Fig. 4:**
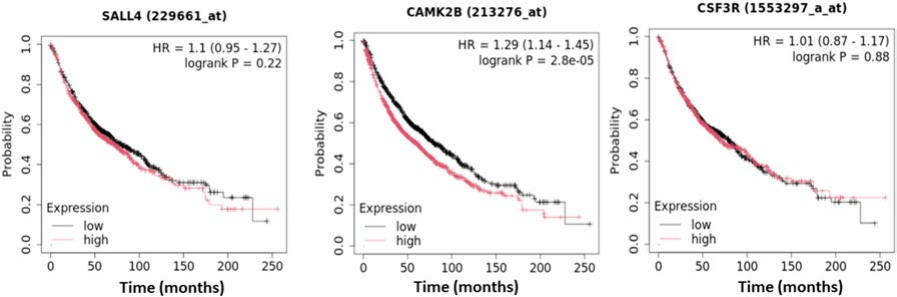
Kaplan–Meier survival analysis of top three significantly dysregulated genes (*SALL4, CAMK2B, CSF3*) common between sarcoidosis and lung cancer

While the violin plot analysis indicated how the expression of the twelve common DEGs varied across different LC stages, we further evaluated their potential clinical relevance by performing overall survival analysis. Similar to the stage-based analysis, the survival analysis was conducted exclusively for LC patients, as survival data were only available for this group. Figure 4 presents the HR with 95% confidence intervals (CI) and log-rank *p*-values for the top three significantly associated genes in these patients. Overall survival analysis using the Kaplan–Meier Plotter indicated that higher expression of *CAMK2B* was significantly associated with poor prognosis in LC patients (HR = 1.29, 95% CI: 1.14–1.25, log-rank p = 2.8e-5). In contrast, *SALL4* (HR = 1.1, 95% CI: 0.95–1.27, log-rank p = 0.22) and *CSF3* (HR = 1.01, 95% CI: 0.87–1.17, log-rank p = 0.88) did not show statistically significant associations with survival outcomes.

To provide a comprehensive overview, Supplementary Fig. 4 illustrates the survival patterns of the remaining analyzed genes, further supporting these findings. The survival impact of *OLR1* (HR = 0.86, 95% CI: 0.76–0.96, log rank *p* = 0.01), *WNT10A* (HR = 0.86, 95% CI: 0.74–0.99, log rank *p* = 0.039), *RASAL1* (HR = 1.25, 95% CI: 1.11–1.41, log rank *p* = 2e-04), *RAMP3* (HR = 0.98, 95% CI: 0.87–1.11, log rank *p* = 0.8), *GADD45B* (HR = 0.87, 95% CI: 0.77–0.98, log rank *p* = 0.02), *KLF4* (HR = 1.15, 95% CI: 1.02–1.3, log rank *p* = 0.02), *AGER* (HR = 0.93, 95% CI: 0.82–1.04, log rank *p* = 0.21), *WIF1* (HR = 0.71, 95% CI: 0.63–0.8, log rank *p* = 1.1e-08), and *PRKAG3* (HR = 1.12, 95% CI: 0.97–1.3, log rank *p* = 0.12) is depicted in Supplementary Fig. 4.

### Interaction network and enriched pathways

**Fig. 5:**
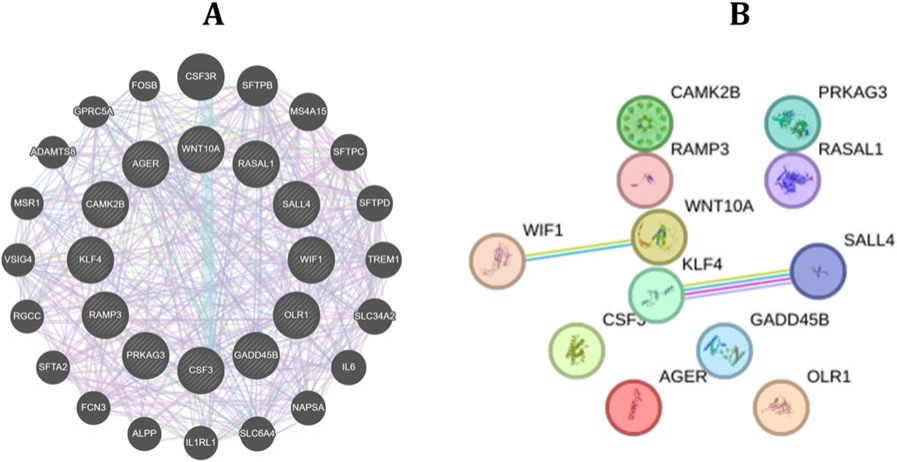
Interaction network analysis of common genes between sarcoidosis and lung cancer. **A** GeneMania interaction network revealed 68.30% co-expression among the overlapping genes, with co-localization observed in 18.64% of cases. **B** STRING tool-based protein–protein interaction (PPI) network showed 12 nodes and 2 edges, with a PPI enrichment *p*-value of 0.22, indicating modest interaction among the common proteins

GeneMania interaction network analysis revealed 68.30% co-expression among these overlapping genes between sarcoidosis and LC. Next, co-localization among these common genes was found to be 18.64% (Fig. 5A). Further details of the interaction network analysis, including individual gene relationships, are provided in Supplementary Table 3. STRING database was used to construct the PPI network, revealing 12 nodes and 2 edges among the proteins encoded by the common genes. The PPI enrichment *p*-value was 0.22, indicating that the observed connectivity was not statistically significant compared to random expectations (Fig. 5B). Next, the pathway enrichment analysis was conducted using Enrichr, and statistical significance was assessed using Fisher’s exact test to account for multiple comparisons. Wnt signaling pathway was found to be significantly enriched in both sarcoidosis and LC, based on the twelve common DEGs between sarcoidosis and LC. The complete list of significantly enriched pathways, along with their corresponding FDR values, is provided in Supplementary Table 4.

### Identification of potential miRNAs and transcription factors

The miRNAs and transcription factors which are associated with the overlapping genes between sarcoidosis and LC was also determined. Two miRNAs, hsa-mir-34a-5p and hsa-mir-16-5p, showed the highest association with these twelve genes based on the degree centrality of the gene-miRNA network (cut-off value = 8) (Supplementary Fig. 5). The complete network comprised 71 nodes and 167 edges. Additionally, the miRNet tool showed three transcription factors (PPARG, NFKB1, and RELA) exhibited the highest levels of interaction with the common DEGs shared between sarcoidosis and LC. These transcription factors were identified based on a degree centrality cut-off value of 2, indicating their potential regulatory significance within the shared gene network (Supplementary Fig. 6).

### WebCSEA demonstrated that sarcoidosis and LC are enriched in epithelial cells

**Fig. 6:**
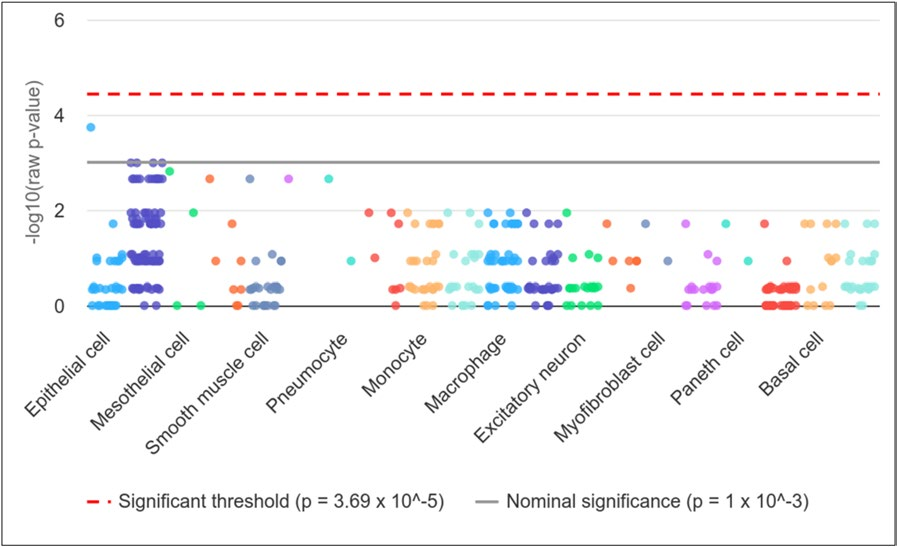
Enrichment analysis using the Web-based Cell-type Specific Enrichment Analysis (WebCSEA) tool revealed epithelial cells as the top enriched immune cells associated with the common genes between sarcoidosis and lung cancer

In this study, WebCSEA tool was employed to investigate the cell enrichment specificity of overlapping genes identified between sarcoidosis and LC. The analysis revealed epithelial cells as the top enriched cell types associated with these common genes (Fig. 6). Epithelial cells serve as the first line of defence in the lung, maintaining barrier integrity and modulating immune responses. In sarcoidosis, granulomatous inflammation disrupts epithelial homeostasis, while in LC, epithelial cells undergo malignant transformation, both contributing to disease progression through fibrosis, immune activation, and tumor development.

### Identification and structural analysis of potential drug

To identify potential therapeutic candidates targeting the twelve common genes implicated in sarcoidosis and LC, we utilized the Drug-Gene Interaction Database (DGIdb) tool. The list of all potential drugs that target the common genes between sarcoidosis and LC is provided in the Supplementary Table 5. Among the drugs analyzed, metformin emerged as the most promising candidate, with an interaction score of 1.795 with *PRKAG3*.

### XRD pattern analysis employing Rietveld refinement for metformin

**Fig. 7:**
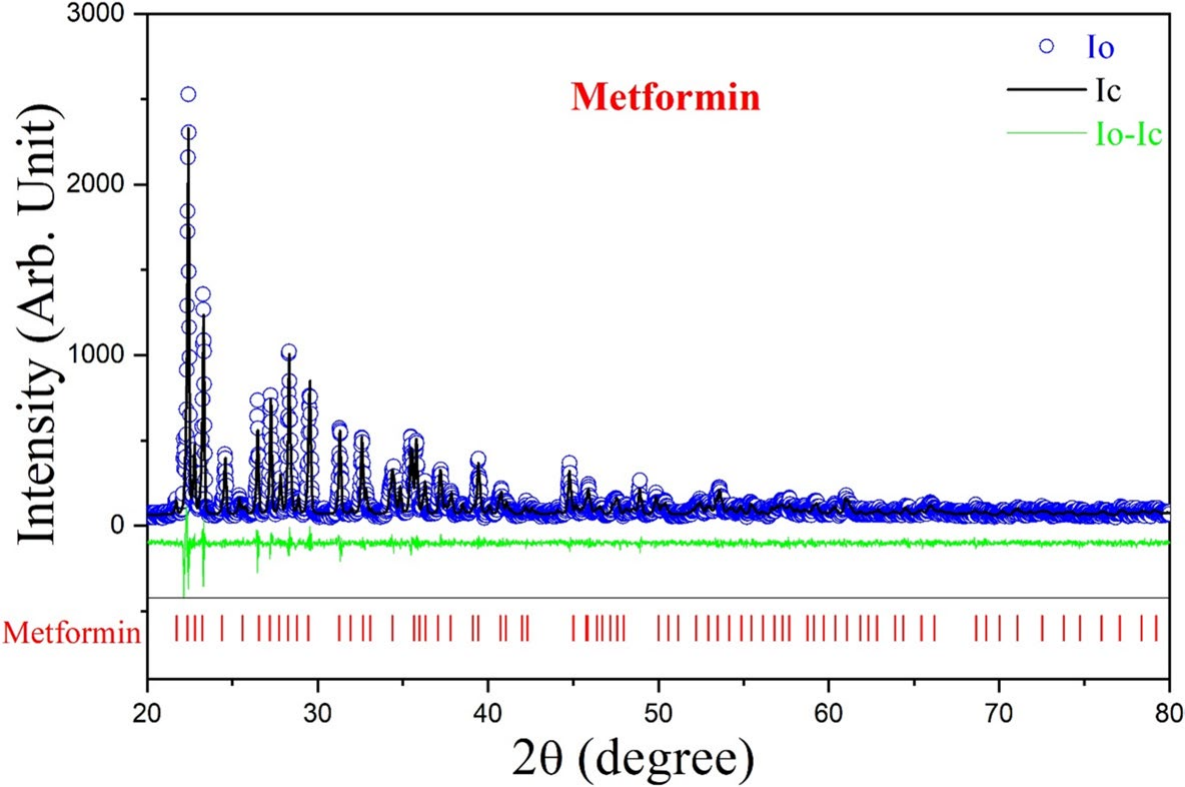
Rietveld refinement of the X-ray diffraction (XRD) pattern of pure metformin, showing the crystalline structure and phase purity of the drug based on XRD data

The structural properties of the candidate drug i.e., metformin was analyzed using XRD, and the resulting pattern was refined through Rietveld analysis to reveal detailed information about its crystalline structure and phase purity. Figure 7 illustrates the Rietveld refinement of the XRD pattern for pure metformin (C₄H₁₂ClN₅), with no observable impurities. The XRD pattern corresponds to a monoclinic crystal system (COD #2,108,029; Space Group: P21/c) with lattice parameters a = 7.9104 Å, b = 13.8794 Å, c = 7.9310 Å, and β = 114.6060°. The alignment of the observed (Io) and computed (Ic) intensities, indicated by hollow blue dots and solid black lines, respectively, demonstrates the accuracy of the refinement. The residuals (Io-Ic), shown as a green line, confirm a high-quality fit.

**Fig. 8:**
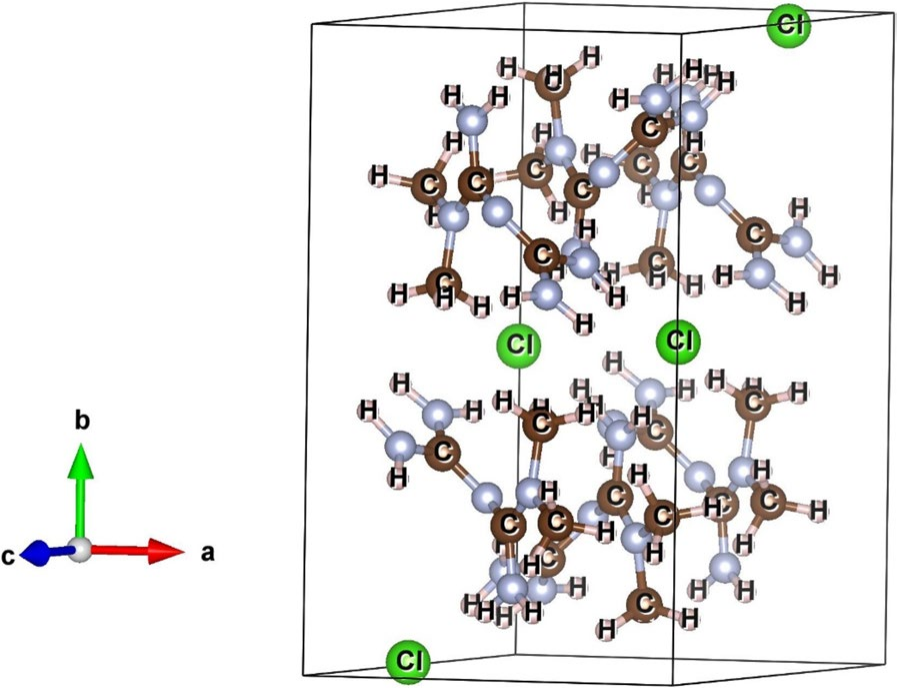
Unit cell structure of metformin as determined from XRD analysis, showing the detailed crystallographic arrangement of the drug molecules

The Rietveld refinement provided crucial structural parameters such as crystallite size and r.m.s. microstrain, which are summarized in Table 2. The results indicate that the pure phase of metformin is composed of nanocrystals approximately 105 nm in size, with minimal lattice strain, supporting the drug's structural integrity. Additionally, the unit cell of metformin was visualized using the VESTA software (Fig. 8), providing a clear representation of its molecular arrangement. These findings confirm that the metformin used in subsequent cell culture assays maintains high structural purity and integrity, supporting its suitability for biological evaluation.

**Table 2:** Structural and microstructural parameters obtained from the Rietveld analysis of metformin

Nano composite	Crystalline compound	Cell parameters (Ȧ)			Crystallite size (nm)	R.m.s Micro Strain (× 10^–4^)
		a	b	c		
Metformin	C_4_H_11_N_5_ • HCl	7.9853	13.9265	7.9852 β = 114.89^0^	104.65	0.11

### Effect of metformin on A549, HeLa and WI38 cells

**Fig. 9:**
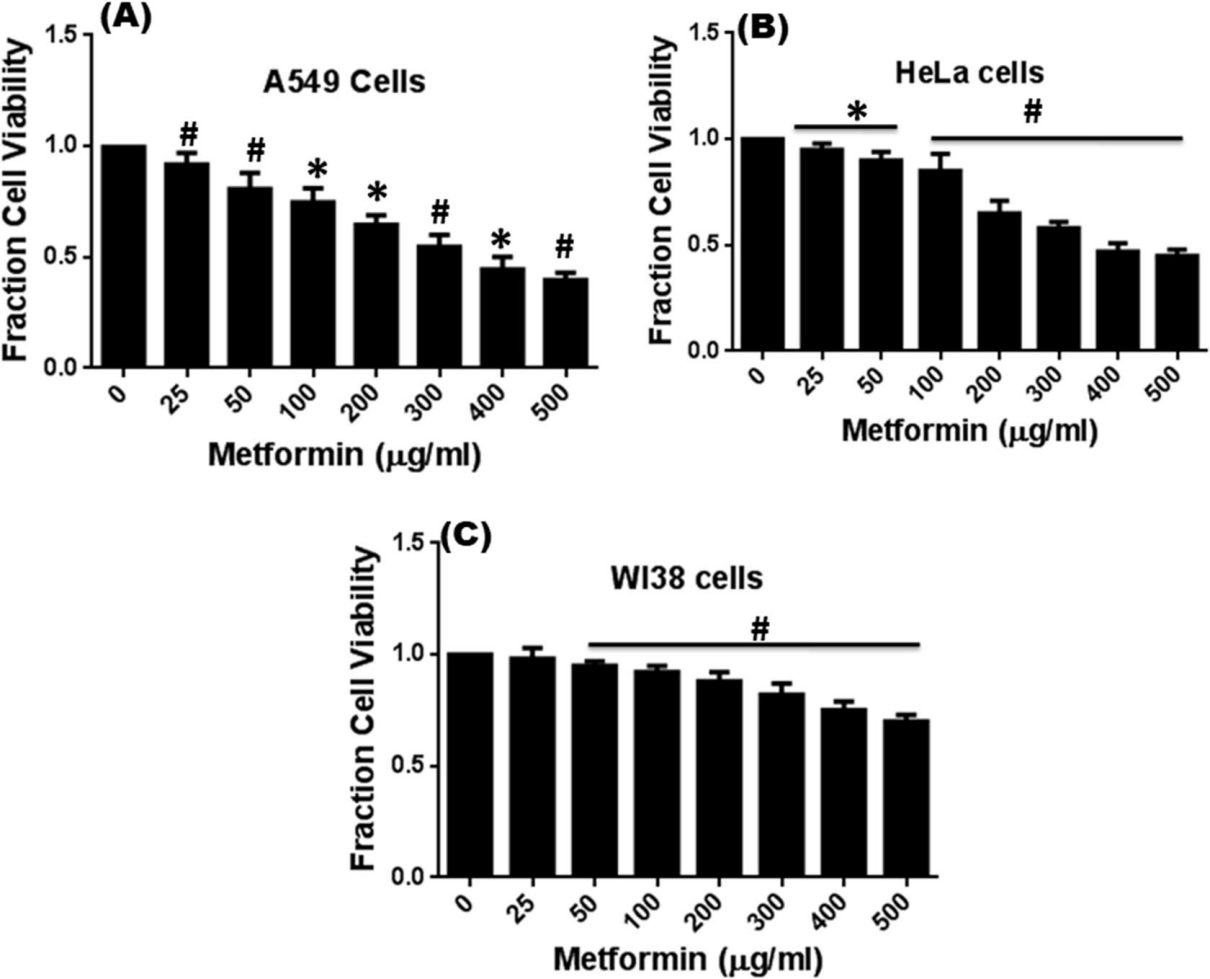
Effect of Metformin on viability of human normal lung carcinoma A549 cells (**A**) and human cervical carcinoma HeLa cells (**B**) and normal lung fibroblast cells WI38 (**C**) by MTT assay after 48 h of treatment. Data represents Mean ± SD of three experimental sets under similar conditions. * *p* <.05 and # *p* <.01 compared to untreated control

*MTT assay*: Metformin reduced viability of both A549 and HeLa cells within 48 h of incubation in concentration dependent fashion with IC50 doses around 350 µg/ml and 400 µg/ml respectively. We also studied the effect of metformin on growth inhibition of normal lung fibroblast WI38 cells under similar growth conditions. But growth inhibition of WI38 was significantly low compared to that of A549 and HeLa cells. We found only 25% cell death maximally after 48 h of treatment with highest dose of metformin on normal lung fibroblasts WI38 cells (500 µg/ml) (Fig. 9). These results suggest that metformin exhibits selective cytotoxicity toward cancer cells while remaining comparatively non-toxic to normal human cells.

### Cell morphology study

**Fig. 10:**
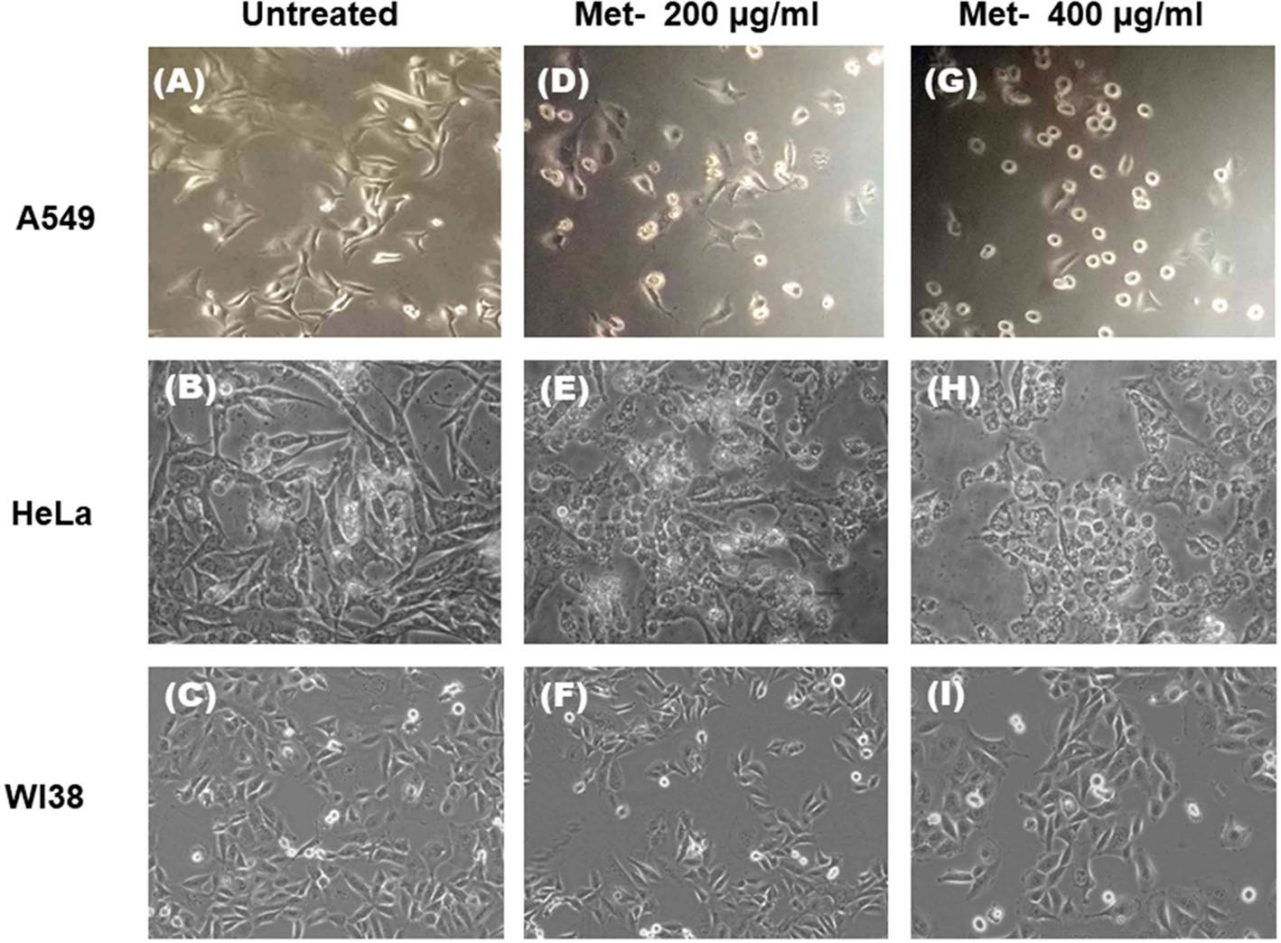
Alteration of morphology of A549, HeLa and WI38 Cells by Metformin. Bright field images of untreated and treated cells were taken by Olympus inverted microscope model CKX41. **A**, **B**, and **C** represent untreated A549, HeLa and WI38 cells respectively. **D**, **E** & **F** represent A549, HeLa and WI38 cells treated with 200 µg/ml metformin and **G**, **H** & **I** represent A549, HeLa and WI38 cells treated with 400 µg/ml metformin. Images were taken under 20X objective lens

We have also observed the morphology change of cancer cells in presence of metformin. Cultured A549, HeLa and WI38 cells were treated with different concentrations of metformin (0, 200 and 400 µg/ml) for 48 h and bright field images of control and treated cells were taken by Olympus inverted microscope model CKX41 (Fig. 10). The left panel in the Fig. 10 (A-C) shows the untreated control cells. At 200 µg/ml dose (below IC50 dose) both A549 and HeLa cells were affected as many cells became smaller in size and round shaped (Fig. 10 D-F). At IC50 dose (400 µg/ml) the morphology of most of the cells was changed and a significant number of cells were totally rounded up and detached from the substratum indicating dead cells (Fig. 10 G-I). WI38 cells, on the other hand, showed more resistant property and were affected less compared to A549 and HeLa cells. Morphology change of WI38 cells was not very profound even at 400 µg/ml dose. This result corroborated well with the result obtained in the MTT assay.

### Colony formation assay

**Fig. 11:**
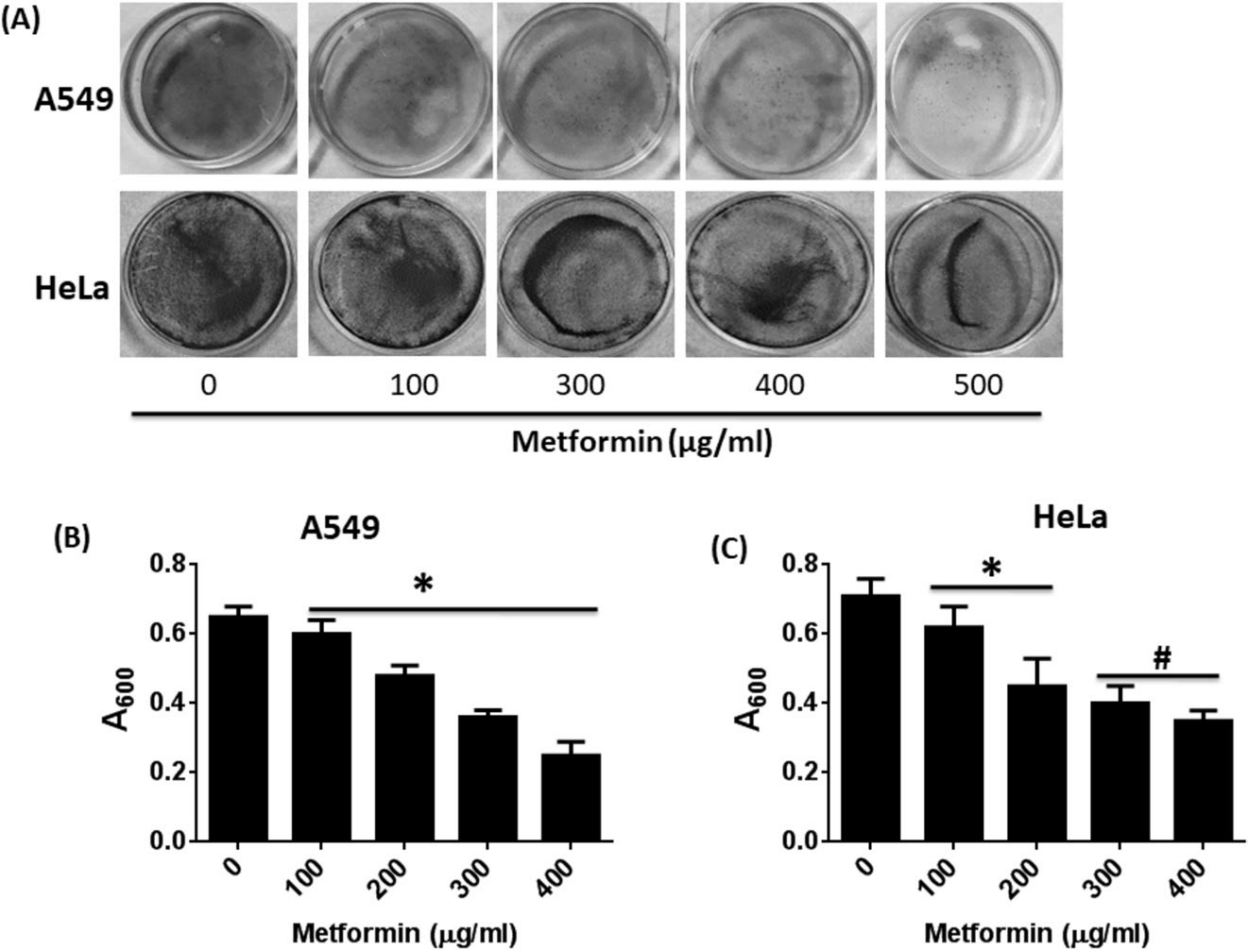
Colony formation assay with A549 and HeLa cells upon metformin treatment. **A** Petri plates showing formation of micro colonies of metformin treated A549 cells and HeLa cells after staining with crystal violet. Images are shown in grayscale mode. **B**, **C** Bar diagram showing the absorbance of crystal violet stain at 600 nm, which is representative of viable colonies formed after metformin treatment. Data represents Mean ± SD of three experimental sets under similar conditions. * *p* <.05 and # *p* <.01 compared to untreated control

To investigate the long-term and irreversible growth inhibitory effect of metformin on A549 and HeLa cells, we observed colony formation of cells after incubating in presence of different concentrations of metformin (0–400 µg/ml) for 48 h and allowing them to form viable colonies for the next seven days. The viable colonies were stained with crystal violet. We found that colony formation of both cell lines was reduced upon metformin treatment in dose-dependent manner (Fig. 11A). The stain was further extracted in 10% acetic acid and the absorbance at 600 nm was taken. A600 was also decreased upon metformin treatment (Fig. 11B & C). The result signifies that colony formation of A549 and HeLa cells was inhibited by metformin and that the compound might have a long-term effect on the growth potential of viable cells.

## Discussion

The progression of sarcoidosis to LC is well documented, and this correlation is significantly associated with poor prognosis in patients with LC. Although the association between these two conditions is frequently observed, a paucity of studies focus on the common genetic overlaps between these two disorders. In contrast to previous studies that emphasized cell type-specific expression patterns, such as plasma cell-related genes in COVID-19 [39] or epigenetically driven gene signatures for cancer prognosis [40], the present study adopts an integrative, systems-level approach. This approach not only strengthens the biological plausibility of the findings but also facilitates the identification of repurposable therapeutic candidates, such as metformin, with potential efficacy across inflammatory and oncogenic contexts. Here we used advanced computational biology-based approach to investigate the correlation between sarcoidosis and LC, identifying overlapping genes, enriched immune cells and common pathways. The expression of these candidate genes was also validated in additional datasets. In addition to these common DEGs, this study also suggests genetic regulators, including miRNAs and transcription factors, and identifies potential drugs targeting the common key genetic signature. Furthermore, detailed structural analysis for the potential drug was employed to investigate the precise structural information about the drug at the atomic level. Finally, the effect of the drug is evaluated in vitro using both cancerous and normal cell lines.

This study indicates that the dysregulation of *SALL4, WNT10A, RASAL1, CAMK2B, GADD45B, KLF4, OLR1, CSF3, WIF1, RAMP3, AGER* and *PRKAG* in both sarcoidosis and LC, which could be attributed to common pathological processes such as chronic inflammation, immune imbalance, and aberrant cellular signaling. For instance, the upregulation of *WNT10A*, *RASAL1*, *CAMK2B*, and *SALL4* suggests their involvement in tumor-promoting processes, such as cell proliferation, migration, and immune evasion [36, 41]. Elevated expression of these genes correlated with poorer survival outcomes in LC patients, reinforcing their potential role in disease progression. Notably, *WNT10A* is a key component of the Wnt/β-catenin signaling pathway, which was found to be significantly enriched in both sarcoidosis and LC (FDR-adjusted *p* = 0.001). Dysregulation of Wnt signaling has been associated with uncontrolled epithelial proliferation, immune evasion, and poor prognosis in LC [42]. Conversely, the alteration of *PRKAG3* in both diseases is likely linked to its role in regulating cellular energy metabolism and stress responses, critical processes that are frequently disrupted in chronic inflammatory conditions and cancer [43]. The coordinated downregulation of *KLF4*, *OLR1*, *CSF3*, *WIF1*, *RAMP3*, and *AGER*, may impair tumor-suppressive mechanisms, thereby facilitating cancer progression, including enhanced tumor growth, migration, invasion, and metastasis [41, 44]. Additionally, *GADD45B* functions as a tumor suppressor by mediating DNA damage response, and its significant downregulation in both diseases suggest impaired cellular stress responses, which may contribute to chronic inflammation in sarcoidosis and tumorigenesis in LC [45]. Among the twelve overlapping genes identified, *SALL4*, *CAMK2B*, and *CSF3R* emerged as the most prominent based on their fold change values, warranting deeper investigation. Overall, the altered expression of these twelve genetic signatures may contribute to the pathogenesis of both sarcoidosis and LC; however, further experimental validation is recommended to confirm this association.

Two microRNAs, hsa-miR-34a-5p and hsa-miR-16-5p, showed strong associations with these twelve common DEGs shared between sarcoidosis and LC, likely due to their regulatory roles in key biological pathways related to inflammation, cell proliferation, and apoptosis. MiR-34a-5p is known to suppress tumor growth and inflammation by targeting genes involved in these processes, thereby potentially influencing the progression of both diseases [46]. Similarly, miR-16-5p has been implicated in regulating cell cycle progression and apoptosis, suggesting its role in modulating cellular responses that are perturbed in both sarcoidosis and cancer [47]. Regarding transcription factors, PPARG, NFKB1, and RELA exhibited the highest level of interaction with the common DEGs in both diseases. Since their involvement in immune response modulation and gene transcription is well-established, these observations appear consistent with known biological functions [48, 49].

It was also found that epithelial cells are enriched among the common genes shared between sarcoidosis and LC. The enrichment of epithelial cells suggests that these cells play a crucial role in the pathogenic processes underlying both diseases. Epithelial cells are major contributors to inflammation, fibrosis, and tissue remodeling, suggesting their involvement in sarcoidosis pathogenesis, where these processes play a central role in disease progression [50, 51]. Similarly, in LC, epithelial cells can undergo malignant transformation due to dysregulated signaling pathways such as Wnt/β-catenin, leading to uncontrolled proliferation and metastasis [52]. Wnt signalling pathway, a crucial regulator of cell proliferation, differentiation, and migration, has been also implicated in the pathogenesis of both sarcoidosis and LC. In sarcoidosis, an inflammatory disease characterized by granuloma formation in the lungs, aberrant Wnt signalling may contributes to the dysregulated immune response and fibrosis [53]. Similarly, in LC, this pathway is often dysregulated, leading to uncontrolled cell growth and metastasis [52]. Key components of the Wnt pathway, such as β-catenin, are often overexpressed or mutated in these conditions, suggesting a potential shared molecular mechanism that could be targeted for therapeutic intervention. Given the strong enrichment of epithelial cells and Wnt signaling in both diseases, further experimental validation—including RT-qPCR, immunohistochemistry, and single-cell transcriptomics—will be essential to confirm these findings and their relevance for precision medicine.

The study further suggests that metformin could serve as a potential therapeutic option for LC patients who either developed the disease following sarcoidosis or present with a co-occurring condition, as mentioned in different studies [3, 4]. Metformin was selected as the potential therapeutic candidate despite its primary target, *PRKAG3*, showing no statistically significant association with survival in LC, as its utility is supported by multiple converging factors. Mechanistically, metformin modulates the Wnt signaling pathway, which was significantly enriched in both sarcoidosis and LC in our analysis, through both AMPK-dependent and AMPK-independent mechanisms [54, 55]. Wnt signaling contributes to fibrosis, a major factor in sarcoidosis-related mortality [56], and metformin has been shown to suppress Wnt/β-catenin activity in LC [57], indicating its relevance in both diseases. Compared to the other identified drug candidates (as shown in Supplementary Table 4), metformin offers a combination of anti-inflammatory and anti-tumorigenic effects, along with oral bioavailability, low toxicity, and a well-established clinical safety profile. It also demonstrated a strong DGIdb interaction score (≥ 1.0) with *PRKAG3*. Literature further supports its use, with studies linking metformin to reduced LC risk and improved patient survival [58]. Furthermore, metformin influences epithelial–mesenchymal transition (EMT) by downregulating transcription factors like KLF4, which was significantly altered in our dataset [59], consistent with our WebCSEA findings that identified epithelial cells as a key enriched cell type. Experimental validation through MTT cytotoxicity assays and XRD with Rietveld refinement further supports the selection of metformin as the potential drug candidate for treating the overlapping condition of sarcoidosis and LC involving the identified shared genetic alterations.

The detailed structural analysis of metformin through XRD analysis, confirming the monoclinic phase and nanocrystalline form (~ 105 nm), further supports its stability and bioavailability. Next, the concentration-dependent reduction in viability of A549 cells suggests that metformin effectively targets cancer cell proliferation. In contrast, the minimal growth inhibition observed in WI38 cells indicates a selective cytotoxicity of metformin towards cancer cells, sparing normal lung fibroblasts. This differential effect underscores the potential of metformin as a therapeutic agent with reduced toxicity to normal tissues. Previous research has demonstrated that metformin induces cytotoxic effects in A549 and HeLa cells through mechanisms such as mitochondrial damage, reactive oxygen species (ROS) generation, activation of oxidative stress pathways, and inhibition of the PI3K/Akt/mTOR signaling pathway. These studies provide additional context for understanding metformin’s selective impact on cancer cells while sparing normal lung fibroblasts [60–64]. Given its ability to interact with multiple biological targets and its well-documented safety profile, metformin holds promise as an effective therapeutic agent in mitigating disease progression in patients exhibiting overlapping features of sarcoidosis and LC, warranting further clinical investigation.

This study has several limitations. First, a deeper exploration of the unique interactions and pathways in sarcoidosis and LC could provide valuable insights into their shared pathophysiology, potentially leading to more targeted and effective therapeutic strategies. Future research should prioritize rigorous experimental validation to substantiate bioinformatics findings and further investigate their therapeutic implications. Second, the expression of *SALL4* and *CAMK2B* did not show statistically significant differences across LC stages. This lack of stage-specific expression dynamics may indicate that these three genes act as early drivers of tumorigenesis, whose dysregulation initiates or supports malignant transformation but is not further modulated during disease progression. Moreover, the progression of LC is influenced by complex and heterogeneous processes—including epigenetic regulation, stromal interactions, immune evasion, and metastatic mechanisms—that may not be directly reflected in gene expression profiles alone. Hence, these genes may serve as stage-independent prognostic indicators, highlighting their potential relevance for early detection or as baseline biomarkers rather than markers of tumor advancement. Third, epithelial cells were identified as the top enriched cell type through cell-type enrichment analysis; a limitation of this study is that epithelial cells were not directly selected as the primary research subject. Instead, we employed A549 cells, an alveolar epithelial carcinoma line representing alveolar epithelial type II cells [65], which is widely used for investigating cytotoxicity, oxidative stress, and (pro-)inflammatory responses [66]. In addition, HeLa cells were included in this study as a well-established epithelial cancer cell line to further support the evaluation of epithelial-specific drug effects. While A549 or HeLa cells retain key epithelial features, they do not fully capture the complexity of native epithelial tissues. Future research will address this limitation by utilizing primary bronchial epithelial cells and 3D epithelial organoid cultures to establish a more physiologically relevant model for investigating epithelial-specific gene regulation and drug response in the context of sarcoidosis and LC. Additionally, the inclusion of NCI-H2087 and other adenocarcinoma-specific cell lines will enhance disease specificity and provide a more comprehensive understanding of drug efficacy across different LC subtypes. Fourth, although the study benefits from the use of multiple publicly available datasets and implements robust statistical corrections, the inherent limitations of in silico analyses remain. Validation in independent patient cohorts, along with RT-qPCR and functional assays, will be essential to confirm biological significance and clinical applicability. Fifth, expanding the analysis to larger patient cohorts is essential to enhance the reliability and generalizability of the findings. While multiple independent transcriptomic datasets strengthened the analysis, further in vivo validation is necessary. Although MTT assays and XRD analysis confirmed metformin’s cytotoxic effects and structural integrity, its long-term impact on tumor progression, immune response, and fibrosis requires validation in animal models and patient-derived xenografts. Future studies can also incorporate RNA-seq and proteomic profiling to better understand metformin’s molecular mechanisms, bridging the gap between bioinformatics-driven discoveries and clinical applications in sarcoidosis and LC. Finally, our study was not specifically designed to investigate a causal transition from sarcoidosis to LC, it aimed to identify shared molecular dysregulation that may contribute to the increased susceptibility of sarcoidosis patients to LC. The therapeutic evaluation of metformin was therefore intended to explore its potential for targeting this common pathogenic axis, rather than treating one condition in isolation. While metformin was found to interact with *PRKAG3*, a gene significantly altered in both conditions, and exhibited cytotoxic effects on LC cells, the extent to which it might prevent malignant progression in high-risk sarcoidosis patients remains to be identified. Future studies should aim to evaluate metformin’s role in early intervention, ideally using longitudinal models or patient-derived systems, to determine whether it can modulate inflammatory or oncogenic processes before full disease onset. This line of investigation will help define whether metformin is more effective as a preventive strategy in sarcoidosis patients predisposed to LC, or as a therapeutic agent in established LC. The current findings, however, highlight its potential dual applicability, reinforcing the value of drug repurposing strategies in targeting converging molecular mechanisms, particularly involving *PRKAG3* dysregulation in sarcoidosis LC overlapping conditions.

In summary, this study highlights the clinical relevance of understanding the relationship between sarcoidosis and LC, particularly adenocarcinoma. Given that granulomas and nodule formation in sarcoidosis are often associated with LC development and progression, further investigation into their impact on disease trajectory and patient survival could facilitate more personalized treatment strategies. Analysing shared molecular pathways and biomarkers may help clinicians differentiate between benign and malignant lung conditions in sarcoidosis patients, enabling earlier detection and more effective management of LC in this population. Ultimately, these insights could contribute to improved patient outcomes by refining diagnostic approaches and optimizing therapeutic interventions.

## Conclusion

The present study advances current knowledge by identifying twelve DEGs common to both sarcoidosis and LC, suggesting a shared pathogenic mechanism between these diseases. Notably, the enrichment of the Wnt signaling pathway among these genes highlights a crucial molecular link that had not been fully established in previous studies. Additionally, our findings reveal a significant enrichment of epithelial cells among these common genes, highlighting their critical role in barrier function, tissue remodeling, and immune regulation. In sarcoidosis, epithelial cells contribute to granuloma formation through interactions with immune cells, whereas in LC, they play a central role in tumor initiation and progression.

Furthermore, we observed that the altered expression of these common genes correlates with advanced tumor stages in LC and poor survival outcomes, emphasizing their potential as prognostic markers. The identification of metformin as a potential therapeutic agent through the DGIdb provides an innovative drug repositioning strategy, bridging genetic insights with treatment opportunities. The structural validation of metformin using XRD analysis with Rietveld refinement adds a layer of precision to the study. Finally, in vitro studies utilizing A549 cells further explored metformin’s therapeutic potential, reinforcing its relevance as a candidate for repurposing. By integrating gene expression analysis, clinical outcome correlations, and drug repositioning approaches, this study significantly enhances the understanding of shared molecular pathways in sarcoidosis and LC, ultimately advancing precision medicine strategies for these diseases.

## Supplementary Information

Supplementary Material 1

## Data Availability

No datasets were generated or analysed during the current study.
