# Supplementary material for: Genetic overlap between sarcoidosis and lung cancer: a combined in silico and in vitro approach
Source: Hereditas. 2025 Aug 12;162:155. doi: 10.1186/s41065-025-00503-7 (PMC12341126; doi:10.1186/s41065-025-00503-7)
Supplement: Supplementary file 1 — Supplementary Material 1 [file 41065_2025_503_MOESM1_ESM.docx]

**Supplementary file**

**Genetic overlap between sarcoidosis and lung cancer: A combined in silico and in vitro approach**

**Sanjukta Dasgupta^1^*, Moupiya Ghosh^2^, Subhendu Chakrabarty^3,4^,**

**Gopal Chakrabarti^3^, Amlan Das^5^**

^1^Department of Biotechnology, Center for Multidisciplinary Research & Innovations, Brainware University, Barasat, India

^2^ Department of basic Science and Humanities, Institute of Engineering and Management (IEM), Kolkata, University of Engineering and Management, Newtown, Kolkata, West Bengal 700160, India

^3^Department of Biotechnology and Dr. B.C. Guha Centre for Genetic Engineering and Biotechnology, University of Calcutta, Kolkata, West Bengal 700019, India

^4^Department of Microbiology, M.U.C. Women's College, Burdwan, West Bengal 713104, India

^5^ Department of Microbiology, Royal School of Biosciences, The Assam Royal Global University, Assam, 781035, India

**Correspondence:**

*Sanjukta Dasgupta, Brainware University, 398, Ramkrishnapur Rd, near Jagadighata Market, Barasat, Kolkata, West Bengal 700125, India; Email id: sd.bt@brainwareuniversity.ac.in

**Supplementary Table 1: Expression of the common significantly altered genes in patients with sarcoidosis as compared to controls**

| **Genes** | **p adjusted**  **(False Discovery Rate)** | **p value** | **log_2_(fold change)** | **Base mean** |
| --- | --- | --- | --- | --- |
| *AGER* | 7.05E-04 | 2.02E-05 | -3.04102 | 31535.57 |
| *CAMK2B* | 1.61E-03 | 9.16E-05 | 3.453907 | 1018.07 |
| *CSF3* | 3.32E-05 | 4.56E-07 | -3.89196 | 4012.29 |
| *GADD45B* | 6.03E-08 | 2.30E-10 | -3.21348 | 26938.79 |
| *KLF4* | 1.71E-05 | 1.76E-07 | -2.17184 | 8474.06 |
| *OLR1* | 1.78E-05 | 1.93E-07 | -2.1015 | 9162.13 |
| *PRKAG3* | 1.12E-03 | 4.82E-05 | 2.898188 | 1050.96 |
| *RAMP3* | 2.07E-04 | 4.02E-06 | -2.5325 | 7137.44 |
| *RASAL1* | 1.61E-03 | 9.05E-05 | 2.070578 | 1373.42 |
| *WIF1* | 6.12E-05 | 8.64E-07 | -2.29145 | 5260.71 |
| *WNT10A* | 1.12E-03 | 4.87E-05 | 2.646674 | 1176.41 |
| *SALL4* | 1.61E-03 | 9.16E-05 | 3.453907 | 1018.07 |

**Supplementary Table 2: Expression of the common significantly altered genes in patients with lung cancer as compared to controls**

| **Genes** | **p adjusted**  **(False Discovery Rate)** | **p value** | **log_2_(fold change)** | **Base mean** |
| --- | --- | --- | --- | --- |
| *AGER* | 1.95E-12 | 8.63E-15 | -2.90336 | 5906.05 |
| *OLR1* | 5.87E-09 | 6.96E-11 | -2.94797 | 1670.87 |
| *SALL4* | 9.72E-07 | 2.49E-08 | 3.021845 | 331.82 |
| *RASAL1* | 3.47E-06 | 1.10E-07 | 2.336243 | 175.06 |
| *RAMP3* | 3.69E-06 | 1.18E-07 | -2.7013 | 521.31 |
| *PRKAG3* | 1.27E-05 | 5.14E-07 | -1.90055 | 99.05 |
| *WIF1* | 5.97E-05 | 3.29E-06 | -2.30521 | 5133.18 |
| *CSF3* | 8.54E-05 | 5.04E-06 | -3.97905 | 1617.88 |
| *CAMK2B* | 2.46E-04 | 1.82E-05 | 3.829276 | 130.91 |
| *GADD45B* | 2.96E-04 | 2.28E-05 | -1.96862 | 8133.9 |
| *WNT10A* | 8.57E-04 | 8.46E-05 | 2.106844 | 105.84 |
| *KLF4* | 8.82E-04 | 8.78E-05 | -2.12086 | 1772.85 |

**Supplementary Table 3: Co-expression and Co-localization network between the common genes of sarcoidosis and lung cancer**

| **Entity 1** | **Entity 2** | **Weight** | **Network group** |
| --- | --- | --- | --- |
| Co-expression | | | |
| *CSF3* | *OLR1* | 0.016125 | Co-expression |
| *RAMP3* | *CSF3* | 0.014383 | Co-expression |
| *AGER* | *OLR1* | 0.013373 | Co-expression |
| *AGER* | *CSF3* | 0.018206 | Co-expression |
| *AGER* | *RAMP3* | 0.013362 | Co-expression |
| *SFTPB* | *OLR1* | 0.013749 | Co-expression |
| *SFTPB* | *CSF3* | 0.015444 | Co-expression |
| *SFTPB* | *RAMP3* | 0.014288 | Co-expression |
| *SFTPB* | *AGER* | 0.019318 | Co-expression |
| *MS4A15* | *OLR1* | 0.013692 | Co-expression |
| *MS4A15* | *CSF3* | 0.020827 | Co-expression |
| *MS4A15* | *RAMP3* | 0.013652 | Co-expression |
| *MS4A15* | *AGER* | 0.019273 | Co-expression |
| *MS4A15* | *SFTPB* | 0.016906 | Co-expression |
| *SFTPC* | *OLR1* | 0.013661 | Co-expression |
| *SFTPC* | *CSF3* | 0.015182 | Co-expression |
| *SFTPC* | *RAMP3* | 0.014015 | Co-expression |
| *SFTPC* | *AGER* | 0.019301 | Co-expression |
| *SFTPC* | *SFTPB* | 0.022545 | Co-expression |
| *SFTPC* | *MS4A15* | 0.017435 | Co-expression |
| *SFTPD* | *OLR1* | 0.014506 | Co-expression |
| *SFTPD* | *CSF3* | 0.016161 | Co-expression |
| *SFTPD* | *RAMP3* | 0.014862 | Co-expression |
| *SFTPD* | *AGER* | 0.019164 | Co-expression |
| *SFTPD* | *SFTPB* | 0.023624 | Co-expression |
| *SFTPD* | *MS4A15* | 0.0157 | Co-expression |
| *SFTPD* | *SFTPC* | 0.022731 | Co-expression |
| *TREM1* | *OLR1* | 0.01732 | Co-expression |
| *TREM1* | *CSF3* | 0.014619 | Co-expression |
| *TREM1* | *RAMP3* | 0.012517 | Co-expression |
| *TREM1* | *AGER* | 0.017353 | Co-expression |
| *TREM1* | *SFTPB* | 0.020249 | Co-expression |
| *TREM1* | *MS4A15* | 0.01634 | Co-expression |
| *TREM1* | *SFTPC* | 0.019917 | Co-expression |
| *TREM1* | *SFTPD* | 0.020273 | Co-expression |
| *SLC34A2* | *OLR1* | 0.01385 | Co-expression |
| *SLC34A2* | *CSF3* | 0.015505 | Co-expression |
| *SLC34A2* | *RAMP3* | 0.014806 | Co-expression |
| *SLC34A2* | *AGER* | 0.019496 | Co-expression |
| *SLC34A2* | *SFTPB* | 0.024361 | Co-expression |
| *SLC34A2* | *MS4A15* | 0.015608 | Co-expression |
| *SLC34A2* | *SFTPC* | 0.023704 | Co-expression |
| *SLC34A2* | *SFTPD* | 0.025498 | Co-expression |
| *SLC34A2* | *TREM1* | 0.021084 | Co-expression |
| *IL6* | *GADD45B* | 0.015994 | Co-expression |
| *IL6* | *CSF3* | 0.026965 | Co-expression |
| *IL6* | *RAMP3* | 0.013462 | Co-expression |
| *IL6* | *AGER* | 0.014626 | Co-expression |
| *IL6* | *SFTPB* | 0.015392 | Co-expression |
| *IL6* | *SFTPC* | 0.015258 | Co-expression |
| *IL6* | *SFTPD* | 0.015804 | Co-expression |
| *IL6* | *TREM1* | 0.014326 | Co-expression |
| *IL6* | *SLC34A2* | 0.018098 | Co-expression |
| *NAPSA* | *CSF3* | 0.014782 | Co-expression |
| *NAPSA* | *RAMP3* | 0.0153 | Co-expression |
| *NAPSA* | *AGER* | 0.018134 | Co-expression |
| *NAPSA* | *SFTPB* | 0.022059 | Co-expression |
| *NAPSA* | *MS4A15* | 0.015028 | Co-expression |
| *NAPSA* | *SFTPC* | 0.021404 | Co-expression |
| *NAPSA* | *SFTPD* | 0.022903 | Co-expression |
| *NAPSA* | *TREM1* | 0.018941 | Co-expression |
| *NAPSA* | *SLC34A2* | 0.023778 | Co-expression |
| *SLC6A4* | *CSF3* | 0.015177 | Co-expression |
| *SLC6A4* | *RAMP3* | 0.013033 | Co-expression |
| *SLC6A4* | *AGER* | 0.019584 | Co-expression |
| *SLC6A4* | *SFTPB* | 0.019974 | Co-expression |
| *SLC6A4* | *MS4A15* | 0.02186 | Co-expression |
| *SLC6A4* | *SFTPC* | 0.020873 | Co-expression |
| *SLC6A4* | *SFTPD* | 0.018037 | Co-expression |
| *SLC6A4* | *TREM1* | 0.018823 | Co-expression |
| *SLC6A4* | *SLC34A2* | 0.019393 | Co-expression |
| *SLC6A4* | *NAPSA* | 0.017134 | Co-expression |
| *IL1RL1* | *CSF3* | 0.020278 | Co-expression |
| *IL1RL1* | *RAMP3* | 0.016111 | Co-expression |
| *IL1RL1* | *AGER* | 0.017701 | Co-expression |
| *IL1RL1* | *SFTPB* | 0.018095 | Co-expression |
| *IL1RL1* | *SFTPC* | 0.01763 | Co-expression |
| *IL1RL1* | *SFTPD* | 0.017892 | Co-expression |
| *IL1RL1* | *TREM1* | 0.017928 | Co-expression |
| *IL1RL1* | *SLC34A2* | 0.018078 | Co-expression |
| *IL1RL1* | *NAPSA* | 0.024627 | Co-expression |
| *ALPP* | *OLR1* | 0.018091 | Co-expression |
| *ALPP* | *CSF3* | 0.034803 | Co-expression |
| *ALPP* | *RAMP3* | 0.014951 | Co-expression |
| *ALPP* | *AGER* | 0.01884 | Co-expression |
| *ALPP* | *MS4A15* | 0.025343 | Co-expression |
| *ALPP* | *SFTPD* | 0.01567 | Co-expression |
| *ALPP* | *IL1RL1* | 0.025825 | Co-expression |
| *FCN3* | *CSF3* | 0.013886 | Co-expression |
| *FCN3* | *RAMP3* | 0.013715 | Co-expression |
| *FCN3* | *AGER* | 0.018719 | Co-expression |
| *FCN3* | *SFTPB* | 0.021297 | Co-expression |
| *FCN3* | *MS4A15* | 0.017381 | Co-expression |
| *FCN3* | *SFTPC* | 0.021222 | Co-expression |
| *FCN3* | *SFTPD* | 0.020875 | Co-expression |
| *FCN3* | *TREM1* | 0.019183 | Co-expression |
| *FCN3* | *SLC34A2* | 0.022106 | Co-expression |
| *FCN3* | *IL6* | 0.014253 | Co-expression |
| *FCN3* | *NAPSA* | 0.020364 | Co-expression |
| *FCN3* | *SLC6A4* | 0.020876 | Co-expression |
| *FCN3* | *IL1RL1* | 0.017973 | Co-expression |
| *SFTA2* | *CSF3* | 0.014163 | Co-expression |
| *SFTA2* | *RAMP3* | 0.013503 | Co-expression |
| *SFTA2* | *AGER* | 0.019224 | Co-expression |
| *SFTA2* | *SFTPB* | 0.022491 | Co-expression |
| *SFTA2* | *MS4A15* | 0.017016 | Co-expression |
| *SFTA2* | *SFTPC* | 0.022335 | Co-expression |
| *SFTA2* | *SFTPD* | 0.023155 | Co-expression |
| *SFTA2* | *TREM1* | 0.019455 | Co-expression |
| *SFTA2* | *SLC34A2* | 0.023726 | Co-expression |
| *SFTA2* | *NAPSA* | 0.021321 | Co-expression |
| *SFTA2* | *SLC6A4* | 0.021183 | Co-expression |
| *SFTA2* | *IL1RL1* | 0.017186 | Co-expression |
| *SFTA2* | *FCN3* | 0.021166 | Co-expression |
| *RGCC* | *OLR1* | 0.024816 | Co-expression |
| *RGCC* | *AGER* | 0.016908 | Co-expression |
| *RGCC* | *SFTPB* | 0.018124 | Co-expression |
| *RGCC* | *MS4A15* | 0.019313 | Co-expression |
| *RGCC* | *SFTPC* | 0.01857 | Co-expression |
| *VSIG4* | *OLR1* | 0.011589 | Co-expression |
| *VSIG4* | *CSF3* | 0.016111 | Co-expression |
| *VSIG4* | *RAMP3* | 0.012796 | Co-expression |
| *VSIG4* | *AGER* | 0.014567 | Co-expression |
| *VSIG4* | *SFTPB* | 0.014295 | Co-expression |
| *VSIG4* | *MS4A15* | 0.015132 | Co-expression |
| *VSIG4* | *SFTPC* | 0.014327 | Co-expression |
| *VSIG4* | *SFTPD* | 0.014694 | Co-expression |
| *VSIG4* | *TREM1* | 0.0131 | Co-expression |
| *VSIG4* | *SLC34A2* | 0.014569 | Co-expression |
| *VSIG4* | *IL6* | 0.014649 | Co-expression |
| *VSIG4* | *NAPSA* | 0.013168 | Co-expression |
| *VSIG4* | *SLC6A4* | 0.014264 | Co-expression |
| *VSIG4* | *IL1RL1* | 0.014802 | Co-expression |
| *VSIG4* | *ALPP* | 0.016947 | Co-expression |
| *VSIG4* | *FCN3* | 0.014537 | Co-expression |
| *VSIG4* | *SFTA2* | 0.014097 | Co-expression |
| *VSIG4* | *RGCC* | 0.016806 | Co-expression |
| *MSR1* | *CSF3* | 0.024264 | Co-expression |
| *MSR1* | *AGER* | 0.017492 | Co-expression |
| *MSR1* | *SFTPB* | 0.019483 | Co-expression |
| *MSR1* | *MS4A15* | 0.021834 | Co-expression |
| *MSR1* | *SFTPC* | 0.018838 | Co-expression |
| *MSR1* | *SFTPD* | 0.021267 | Co-expression |
| *MSR1* | *ALPP* | 0.033091 | Co-expression |
| *MSR1* | *VSIG4* | 0.017057 | Co-expression |
| *ADAMTS8* | *CSF3* | 0.022879 | Co-expression |
| *ADAMTS8* | *AGER* | 0.019573 | Co-expression |
| *ADAMTS8* | *SFTPB* | 0.01972 | Co-expression |
| *ADAMTS8* | *MS4A15* | 0.019407 | Co-expression |
| *ADAMTS8* | *SFTPC* | 0.019565 | Co-expression |
| *ADAMTS8* | *SFTPD* | 0.021055 | Co-expression |
| *ADAMTS8* | *SLC34A2* | 0.019066 | Co-expression |
| *ADAMTS8* | *ALPP* | 0.026663 | Co-expression |
| *ADAMTS8* | *FCN3* | 0.017251 | Co-expression |
| *GPRC5A* | *CSF3* | 0.021982 | Co-expression |
| *GPRC5A* | *KLF4* | 0.010746 | Co-expression |
| *GPRC5A* | *AGER* | 0.018011 | Co-expression |
| *GPRC5A* | *SFTPB* | 0.017448 | Co-expression |
| *GPRC5A* | *MS4A15* | 0.018669 | Co-expression |
| *GPRC5A* | *SFTPC* | 0.017556 | Co-expression |
| *GPRC5A* | *SFTPD* | 0.017634 | Co-expression |
| *GPRC5A* | *TREM1* | 0.016124 | Co-expression |
| *GPRC5A* | *SLC34A2* | 0.019457 | Co-expression |
| *GPRC5A* | *IL6* | 0.021308 | Co-expression |
| *GPRC5A* | *NAPSA* | 0.016163 | Co-expression |
| *GPRC5A* | *SLC6A4* | 0.01778 | Co-expression |
| *GPRC5A* | *FCN3* | 0.016735 | Co-expression |
| *GPRC5A* | *SFTA2* | 0.020509 | Co-expression |
| *GPRC5A* | *VSIG4* | 0.014999 | Co-expression |
| *FOSB* | *GADD45B* | 0.017658 | Co-expression |
| *FOSB* | *CSF3* | 0.011311 | Co-expression |
| *FOSB* | *KLF4* | 0.010061 | Co-expression |
| *FOSB* | *IL6* | 0.01537 | Co-expression |
| *FOSB* | *GPRC5A* | 0.015204 | Co-expression |
| *RAMP3* | *GADD45B* | 0.008822 | Co-expression |
| *AGER* | *WIF1* | 0.013182 | Co-expression |
| *AGER* | *OLR1* | 0.013171 | Co-expression |
| *CSF3R* | *OLR1* | 0.021353 | Co-expression |
| *SFTPB* | *WIF1* | 0.013689 | Co-expression |
| *SFTPB* | *OLR1* | 0.013671 | Co-expression |
| *SFTPB* | *AGER* | 0.010724 | Co-expression |
| *MS4A15* | *WIF1* | 0.015776 | Co-expression |
| *MS4A15* | *AGER* | 0.011107 | Co-expression |
| *MS4A15* | *SFTPB* | 0.011454 | Co-expression |
| *SFTPC* | *WIF1* | 0.013637 | Co-expression |
| *SFTPC* | *OLR1* | 0.013594 | Co-expression |
| *SFTPC* | *AGER* | 0.010638 | Co-expression |
| *SFTPC* | *SFTPB* | 0.011058 | Co-expression |
| *SFTPC* | *MS4A15* | 0.011408 | Co-expression |
| *SFTPD* | *WIF1* | 0.01362 | Co-expression |
| *SFTPD* | *OLR1* | 0.01348 | Co-expression |
| *SFTPD* | *AGER* | 0.010558 | Co-expression |
| *SFTPD* | *SFTPB* | 0.010972 | Co-expression |
| *SFTPD* | *MS4A15* | 0.011392 | Co-expression |
| *SFTPD* | *SFTPC* | 0.010909 | Co-expression |
| *TREM1* | *AGER* | 0.01216 | Co-expression |
| *TREM1* | *SFTPB* | 0.012676 | Co-expression |
| *TREM1* | *SFTPC* | 0.012597 | Co-expression |
| *TREM1* | *SFTPD* | 0.012497 | Co-expression |
| *SLC34A2* | *WIF1* | 0.015181 | Co-expression |
| *SLC34A2* | *AGER* | 0.011402 | Co-expression |
| *SLC34A2* | *SFTPB* | 0.011772 | Co-expression |
| *SLC34A2* | *MS4A15* | 0.013979 | Co-expression |
| *SLC34A2* | *SFTPC* | 0.011668 | Co-expression |
| *SLC34A2* | *SFTPD* | 0.011651 | Co-expression |
| *NAPSA* | *WIF1* | 0.012725 | Co-expression |
| *NAPSA* | *OLR1* | 0.013021 | Co-expression |
| *NAPSA* | *AGER* | 0.010007 | Co-expression |
| *NAPSA* | *SFTPB* | 0.010411 | Co-expression |
| *NAPSA* | *MS4A15* | 0.010859 | Co-expression |
| *NAPSA* | *SFTPC* | 0.010352 | Co-expression |
| *NAPSA* | *SFTPD* | 0.010273 | Co-expression |
| *NAPSA* | *TREM1* | 0.011861 | Co-expression |
| *NAPSA* | *SLC34A2* | 0.011375 | Co-expression |
| *SLC6A4* | *OLR1* | 0.020564 | Co-expression |
| *SLC6A4* | *AGER* | 0.012462 | Co-expression |
| *SLC6A4* | *SFTPB* | 0.012971 | Co-expression |
| *SLC6A4* | *SFTPC* | 0.012906 | Co-expression |
| *SLC6A4* | *SFTPD* | 0.012807 | Co-expression |
| *SLC6A4* | *TREM1* | 0.015901 | Co-expression |
| *SLC6A4* | *SLC34A2* | 0.01381 | Co-expression |
| *SLC6A4* | *NAPSA* | 0.012115 | Co-expression |
| *IL1RL1* | *OLR1* | 0.026346 | Co-expression |
| *IL1RL1* | *CSF3R* | 0.018612 | Co-expression |
| *IL1RL1* | *SLC6A4* | 0.016878 | Co-expression |
| *FCN3* | *WIF1* | 0.014101 | Co-expression |
| *FCN3* | *OLR1* | 0.014746 | Co-expression |
| *FCN3* | *AGER* | 0.011108 | Co-expression |
| *FCN3* | *SFTPB* | 0.011569 | Co-expression |
| *FCN3* | *MS4A15* | 0.011994 | Co-expression |
| *FCN3* | *SFTPC* | 0.011499 | Co-expression |
| *FCN3* | *SFTPD* | 0.011414 | Co-expression |
| *FCN3* | *TREM1* | 0.013079 | Co-expression |
| *FCN3* | *SLC34A2* | 0.012517 | Co-expression |
| *FCN3* | *NAPSA* | 0.011103 | Co-expression |
| *FCN3* | *SLC6A4* | 0.013567 | Co-expression |
| *SFTA2* | *WIF1* | 0.013398 | Co-expression |
| *SFTA2* | *OLR1* | 0.013345 | Co-expression |
| *SFTA2* | *AGER* | 0.010461 | Co-expression |
| *SFTA2* | *SFTPB* | 0.010872 | Co-expression |
| *SFTA2* | *MS4A15* | 0.011312 | Co-expression |
| *SFTA2* | *SFTPC* | 0.010811 | Co-expression |
| *SFTA2* | *SFTPD* | 0.010725 | Co-expression |
| *SFTA2* | *TREM1* | 0.012376 | Co-expression |
| *SFTA2* | *SLC34A2* | 0.011535 | Co-expression |
| *SFTA2* | *NAPSA* | 0.0102 | Co-expression |
| *SFTA2* | *SLC6A4* | 0.012678 | Co-expression |
| *SFTA2* | *FCN3* | 0.011321 | Co-expression |
| *RGCC* | *WIF1* | 0.009997 | Co-expression |
| *RGCC* | *OLR1* | 0.012263 | Co-expression |
| *RGCC* | *AGER* | 0.007839 | Co-expression |
| *RGCC* | *SFTPB* | 0.008143 | Co-expression |
| *RGCC* | *MS4A15* | 0.008696 | Co-expression |
| *RGCC* | *SFTPC* | 0.008082 | Co-expression |
| *RGCC* | *SFTPD* | 0.00802 | Co-expression |
| *RGCC* | *TREM1* | 0.00953 | Co-expression |
| *RGCC* | *SLC34A2* | 0.008773 | Co-expression |
| *RGCC* | *NAPSA* | 0.007758 | Co-expression |
| *RGCC* | *SLC6A4* | 0.010168 | Co-expression |
| *RGCC* | *IL1RL1* | 0.010671 | Co-expression |
| *RGCC* | *FCN3* | 0.008676 | Co-expression |
| *VSIG4* | *OLR1* | 0.012141 | Co-expression |
| *VSIG4* | *RAMP3* | 0.006362 | Co-expression |
| *VSIG4* | *AGER* | 0.007844 | Co-expression |
| *VSIG4* | *SFTPB* | 0.008152 | Co-expression |
| *VSIG4* | *MS4A15* | 0.0087 | Co-expression |
| *VSIG4* | *SFTPC* | 0.008084 | Co-expression |
| *VSIG4* | *SFTPD* | 0.008014 | Co-expression |
| *VSIG4* | *TREM1* | 0.009611 | Co-expression |
| *VSIG4* | *SLC34A2* | 0.008723 | Co-expression |
| *VSIG4* | *NAPSA* | 0.00776 | Co-expression |
| *VSIG4* | *SLC6A4* | 0.010362 | Co-expression |
| *VSIG4* | *IL1RL1* | 0.011102 | Co-expression |
| *VSIG4* | *FCN3* | 0.008896 | Co-expression |
| *VSIG4* | *SFTA2* | 0.007984 | Co-expression |
| *VSIG4* | *RGCC* | 0.006925 | Co-expression |
| *MSR1* | *WIF1* | 0.015831 | Co-expression |
| *MSR1* | *AGER* | 0.011503 | Co-expression |
| *MSR1* | *SFTPB* | 0.011922 | Co-expression |
| *MSR1* | *SFTPC* | 0.011839 | Co-expression |
| *MSR1* | *SFTPD* | 0.011819 | Co-expression |
| *MSR1* | *NAPSA* | 0.011404 | Co-expression |
| *MSR1* | *FCN3* | 0.01319 | Co-expression |
| *MSR1* | *RGCC* | 0.009644 | Co-expression |
| *MSR1* | *VSIG4* | 0.009696 | Co-expression |
| *ADAMTS8* | *WIF1* | 0.020627 | Co-expression |
| *FOSB* | *IL6* | 0.015674 | Co-expression |
| *WIF1* | *SALL4* | 0.024098 | Co-expression |
| *RAMP3* | *WIF1* | 0.006035 | Co-expression |
| *KLF4* | *RAMP3* | 0.005504 | Co-expression |
| *FCN3* | *KLF4* | 0.007259 | Co-expression |
| *RASAL1* | *WNT10A* | 0.010297 | Co-expression |
| *SLC34A2* | *SFTPD* | 0.01114 | Co-expression |
| *FCN3* | *SFTPC* | 0.009701 | Co-expression |
| *SFTA2* | *IL6* | 0.004882 | Co-expression |
| *FOSB* | *GADD45B* | 0.01559 | Co-expression |
| *AGER* | *WIF1* | 0.011909 | Co-expression |
| *CSF3R* | *AGER* | 0.01144 | Co-expression |
| *SFTPB* | *CSF3R* | 0.010868 | Co-expression |
| *SFTPC* | *WIF1* | 0.008522 | Co-expression |
| *SFTPC* | *AGER* | 0.01215 | Co-expression |
| *SFTPC* | *SFTPB* | 0.009404 | Co-expression |
| *SFTPD* | *CSF3R* | 0.009378 | Co-expression |
| *SFTPD* | *SFTPB* | 0.012921 | Co-expression |
| *SFTPD* | *SFTPC* | 0.008834 | Co-expression |
| *TREM1* | *OLR1* | 0.01478 | Co-expression |
| *TREM1* | *CSF3R* | 0.011892 | Co-expression |
| *SLC34A2* | *SFTPB* | 0.011486 | Co-expression |
| *SLC34A2* | *SFTPC* | 0.008587 | Co-expression |
| *SLC34A2* | *SFTPD* | 0.010825 | Co-expression |
| *RGCC* | *WIF1* | 0.013953 | Co-expression |
| *RGCC* | *AGER* | 0.013884 | Co-expression |
| *RGCC* | *SFTPC* | 0.013643 | Co-expression |
| *MSR1* | *VSIG4* | 0.018592 | Co-expression |
| *FOSB* | *GADD45B* | 0.021121 | Co-expression |
| *FOSB* | *FCN3* | 0.010182 | Co-expression |
| *KLF4* | *GADD45B* | 0.010176 | Co-expression |
| *CSF3R* | *OLR1* | 0.013501 | Co-expression |
| *IL6* | *OLR1* | 0.012882 | Co-expression |
| *IL6* | *GADD45B* | 0.011438 | Co-expression |
| *IL6* | *CSF3R* | 0.012646 | Co-expression |
| *VSIG4* | *GADD45B* | 0.009005 | Co-expression |
| *GPRC5A* | *GADD45B* | 0.01801 | Co-expression |
| *GPRC5A* | *KLF4* | 0.01468 | Co-expression |
| *FOSB* | *GADD45B* | 0.017709 | Co-expression |
| ***Co-localization*** | | | |
| *Entity 1* | *Entity 2* | Weight | Network group |
| *AGER* | *CSF3* | 0.021681 | Co-localization |
| *SFTPB* | *CSF3* | 0.036232 | Co-localization |
| *SFTPB* | *AGER* | 0.03976 | Co-localization |
| *IL6* | *CSF3* | 0.028604 | Co-localization |
| *IL6* | *AGER* | 0.021535 | Co-localization |
| *IL6* | *SFTPB* | 0.028831 | Co-localization |
| *SLC6A4* | *AGER* | 0.021004 | Co-localization |
| *SLC6A4* | *SFTPB* | 0.03473 | Co-localization |
| *AGER* | *OLR1* | 0.013076 | Co-localization |
| *AGER* | *RAMP3* | 0.013133 | Co-localization |
| *SFTPB* | *CSF3* | 0.018629 | Co-localization |
| *SFTPB* | *AGER* | 0.010925 | Co-localization |
| *SFTPD* | *OLR1* | 0.016278 | Co-localization |
| *SFTPD* | *AGER* | 0.010926 | Co-localization |
| *TREM1* | *OLR1* | 0.012886 | Co-localization |
| *TREM1* | *GADD45B* | 0.014333 | Co-localization |
| *TREM1* | *RAMP3* | 0.012606 | Co-localization |
| *TREM1* | *AGER* | 0.009044 | Co-localization |
| *TREM1* | *SFTPD* | 0.012914 | Co-localization |
| *SLC34A2* | *CSF3* | 0.021283 | Co-localization |
| *SLC34A2* | *AGER* | 0.009913 | Co-localization |
| *SLC34A2* | *SFTPB* | 0.022199 | Co-localization |
| *IL6* | *AGER* | 0.009121 | Co-localization |
| *IL6* | *SFTPB* | 0.013961 | Co-localization |
| *IL6* | *SLC34A2* | 0.014605 | Co-localization |
| *NAPSA* | *OLR1* | 0.019433 | Co-localization |
| *NAPSA* | *AGER* | 0.015918 | Co-localization |
| *NAPSA* | *TREM1* | 0.01465 | Co-localization |
| *SLC6A4* | *OLR1* | 0.016074 | Co-localization |
| *SLC6A4* | *RAMP3* | 0.014424 | Co-localization |
| *SLC6A4* | *AGER* | 0.012016 | Co-localization |
| *SLC6A4* | *SFTPB* | 0.01025 | Co-localization |
| *SLC6A4* | *SFTPD* | 0.013273 | Co-localization |
| *SLC6A4* | *TREM1* | 0.01069 | Co-localization |
| *SLC6A4* | *NAPSA* | 0.018241 | Co-localization |
| *IL1RL1* | *CSF3* | 0.017293 | Co-localization |
| *IL1RL1* | *AGER* | 0.009445 | Co-localization |
| *IL1RL1* | *SFTPB* | 0.016231 | Co-localization |
| *IL1RL1* | *SLC34A2* | 0.017025 | Co-localization |
| *ALPP* | *CSF3R* | 0.01046 | Co-localization |
| *FCN3* | *GADD45B* | 0.028701 | Co-localization |
| *FCN3* | *SFTPD* | 0.021284 | Co-localization |
| *FCN3* | *TREM1* | 0.015947 | Co-localization |
| *RGCC* | *OLR1* | 0.025595 | Co-localization |
| *RGCC* | *AGER* | 0.014822 | Co-localization |
| *RGCC* | *TREM1* | 0.014319 | Co-localization |
| *RGCC* | *SLC6A4* | 0.018084 | Co-localization |
| *VSIG4* | *OLR1* | 0.014431 | Co-localization |
| *VSIG4* | *AGER* | 0.010732 | Co-localization |
| *VSIG4* | *TREM1* | 0.009695 | Co-localization |
| *VSIG4* | *SLC6A4* | 0.012948 | Co-localization |
| *VSIG4* | *IL1RL1* | 0.013036 | Co-localization |
| *VSIG4* | *RGCC* | 0.015264 | Co-localization |
| *MSR1* | *OLR1* | 0.014261 | Co-localization |
| *MSR1* | *RAMP3* | 0.013339 | Co-localization |
| *MSR1* | *AGER* | 0.01069 | Co-localization |
| *MSR1* | *SFTPD* | 0.011501 | Co-localization |
| *MSR1* | *TREM1* | 0.009455 | Co-localization |
| *MSR1* | *IL6* | 0.008136 | Co-localization |
| *MSR1* | *NAPSA* | 0.016031 | Co-localization |
| *MSR1* | *SLC6A4* | 0.012932 | Co-localization |
| *MSR1* | *RGCC* | 0.016372 | Co-localization |
| *MSR1* | *VSIG4* | 0.011888 | Co-localization |
| *ADAMTS8* | *OLR1* | 0.020427 | Co-localization |
| *ADAMTS8* | *AGER* | 0.013518 | Co-localization |
| *ADAMTS8* | *TREM1* | 0.013711 | Co-localization |
| *ADAMTS8* | *SLC6A4* | 0.016919 | Co-localization |
| *ADAMTS8* | *VSIG4* | 0.014026 | Co-localization |
| *ADAMTS8* | *MSR1* | 0.014039 | Co-localization |
| *GPRC5A* | *OLR1* | 0.010834 | Co-localization |
| *GPRC5A* | *RAMP3* | 0.015492 | Co-localization |
| *GPRC5A* | *AGER* | 0.009451 | Co-localization |
| *GPRC5A* | *SFTPB* | 0.011879 | Co-localization |
| *GPRC5A* | *TREM1* | 0.008332 | Co-localization |
| *GPRC5A* | *SLC34A2* | 0.01156 | Co-localization |
| *GPRC5A* | *IL6* | 0.011319 | Co-localization |
| *GPRC5A* | *SLC6A4* | 0.010595 | Co-localization |
| *GPRC5A* | *RGCC* | 0.017912 | Co-localization |
| *GPRC5A* | *VSIG4* | 0.009677 | Co-localization |
| *GPRC5A* | *MSR1* | 0.009421 | Co-localization |
| *FOSB* | *OLR1* | 0.015364 | Co-localization |
| *FOSB* | *GADD45B* | 0.021061 | Co-localization |
| *FOSB* | *RAMP3* | 0.021256 | Co-localization |
| *FOSB* | *AGER* | 0.011528 | Co-localization |
| *FOSB* | *SFTPD* | 0.017796 | Co-localization |
| *FOSB* | *TREM1* | 0.011791 | Co-localization |
| *FOSB* | *SLC6A4* | 0.014036 | Co-localization |
| *FOSB* | *RGCC* | 0.018312 | Co-localization |
| *FOSB* | *VSIG4* | 0.014161 | Co-localization |
| *FOSB* | *MSR1* | 0.012639 | Co-localization |
| *FOSB* | *GPRC5A* | 0.014459 | Co-localization |

**Supplementary Table 4: List of enriched pathways associated with the common genes between sarcoidosis and LC**

| **Index** | **Name** | **P-value** | **Adjusted p-value** | **Odds ratio** | **Combined score** |
| --- | --- | --- | --- | --- | --- |
| 1 | Wnt signaling pathway | 0.0001169 | 0.009821 | 40.54 | 367.07 |
| 2 | Lipid and atherosclerosis | 0.0002509 | 0.01054 | 31.09 | 257.79 |
| 3 | Basal cell carcinoma | 0.0006315 | 0.01768 | 65.33 | 481.34 |
| 4 | Glioma | 0.0008938 | 0.01877 | 54.56 | 383.03 |
| 5 | Melanogenesis | 0.001612 | 0.02246 | 40.18 | 258.36 |
| 6 | Glucagon signaling pathway | 0.001807 | 0.02246 | 37.87 | 239.20 |
| 7 | Pathways of neurodegeneration | 0.002496 | 0.02246 | 13.78 | 82.60 |
| 8 | FoxO signaling pathway | 0.002692 | 0.02246 | 30.79 | 182.20 |
| 9 | Signaling pathways regulating pluripotency of stem cells | 0.003197 | 0.02246 | 28.15 | 161.75 |
| 10 | Breast cancer | 0.003374 | 0.02246 | 27.37 | 155.78 |

**Supplementary Table 5: List of potential drugs targeting the common genes between sarcoidosis and lung cancer**

| Genes | Drugs | Regulatory approval |
| --- | --- | --- |
| *PRKAG3* | Metformin | Approved |
| *CSF3* | Efbemalenograstim Alfa | Approved |
| *RAMP3* | Pramlintide | Approved |
| *RAMP3* | Pramlintide acetate | Approved |
| *CSF3* | Eflapegrastim | Approved |
| *AGER* | Vitamin B12 | Approved |
| *KLF4* | Hydroxyurea | Approved |
| *RAMP3* | Streptozocin | Approved |
| *CAMK2B* | Hydrogen peroxide | Approved |
| *CAMK2B* | Argipressin | Approved |
| *CAMK2B* | Trifluoperazine | Approved |
| *CAMK2B* | Caffeine | Approved |
| *CAMK2B* | Dehydrated alcohol | Approved |
| *CAMK2B* | Nifedipine | Approved |
| *CAMK2B* | Sorafenib | Approved |


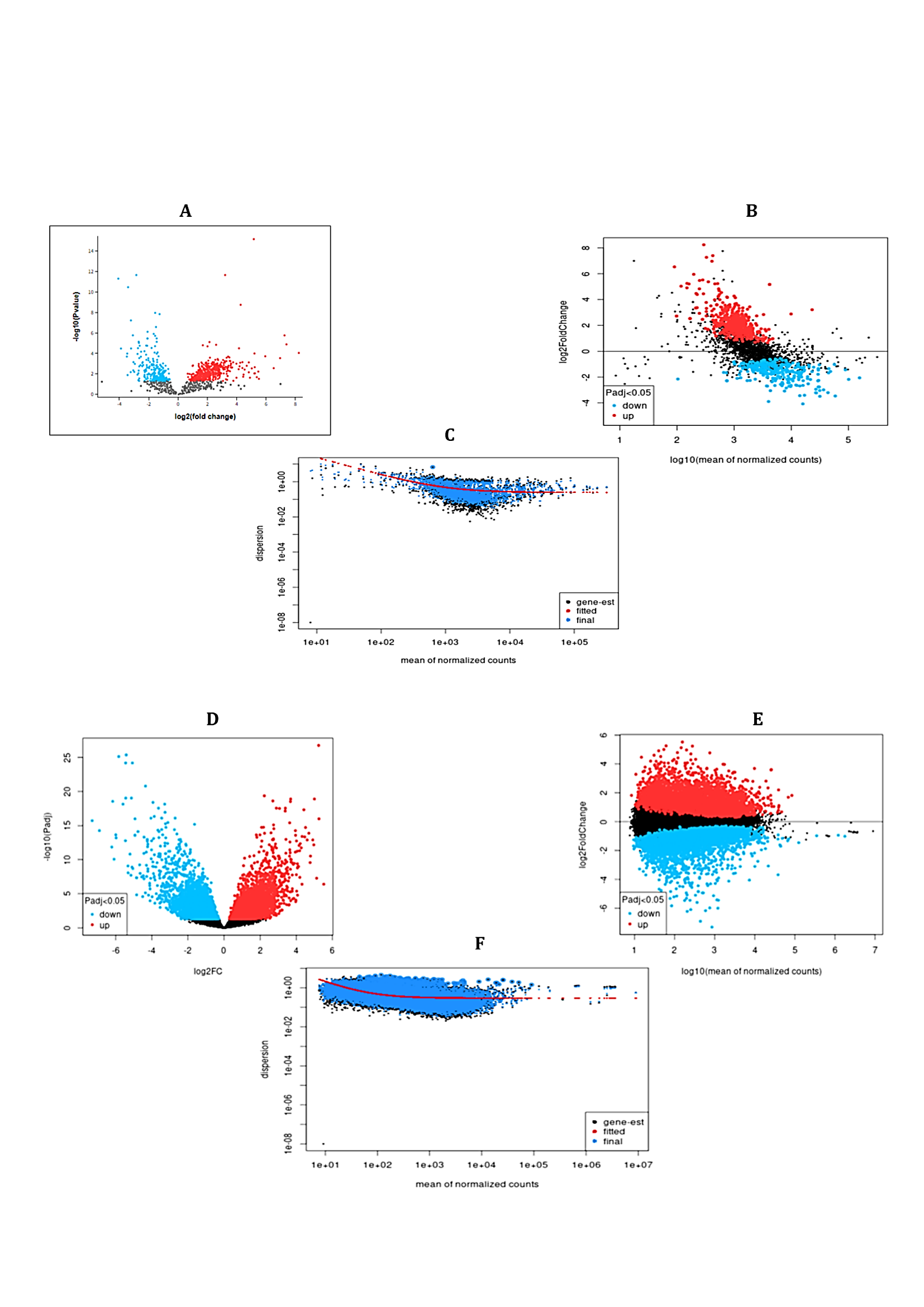


**Supplementary Figure 1:** (A) and (D) Variable importance in projection (VIP), (B) and (E) mean-variance, and (C) and (F) mean dispersion plot of the differentially expressed genes in sarcoidosis and lung cancer as compared to controls.


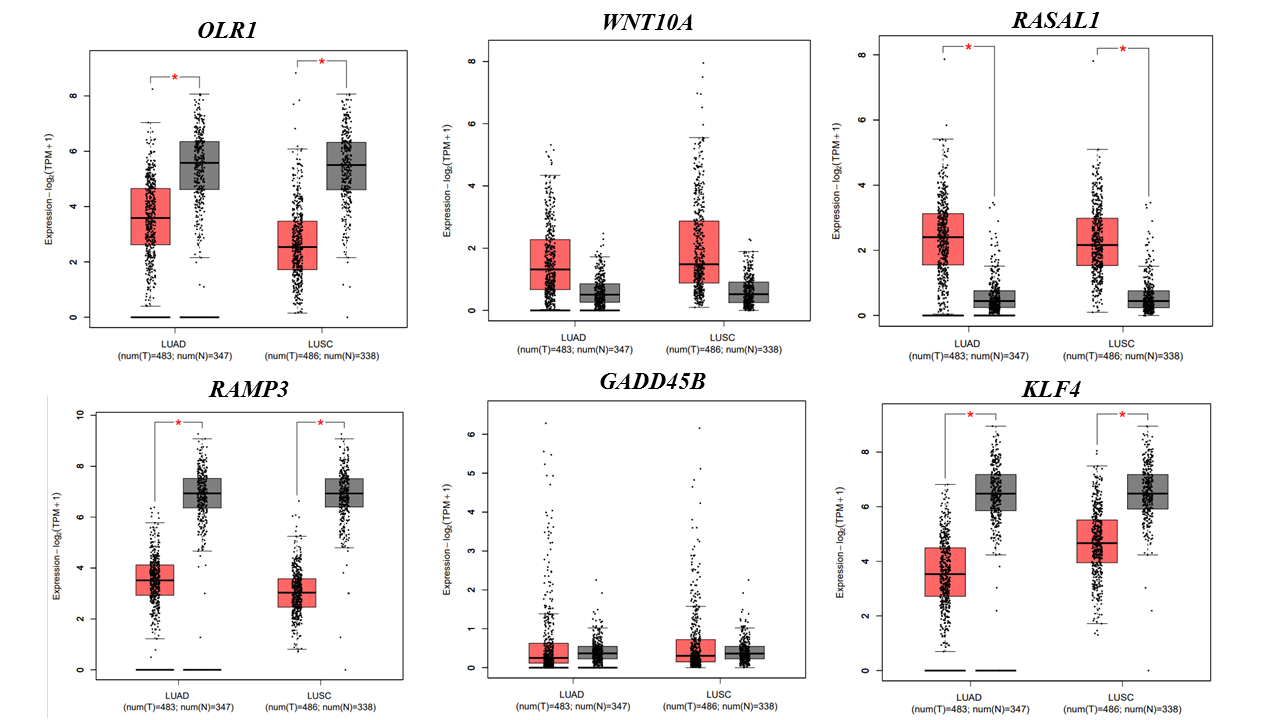


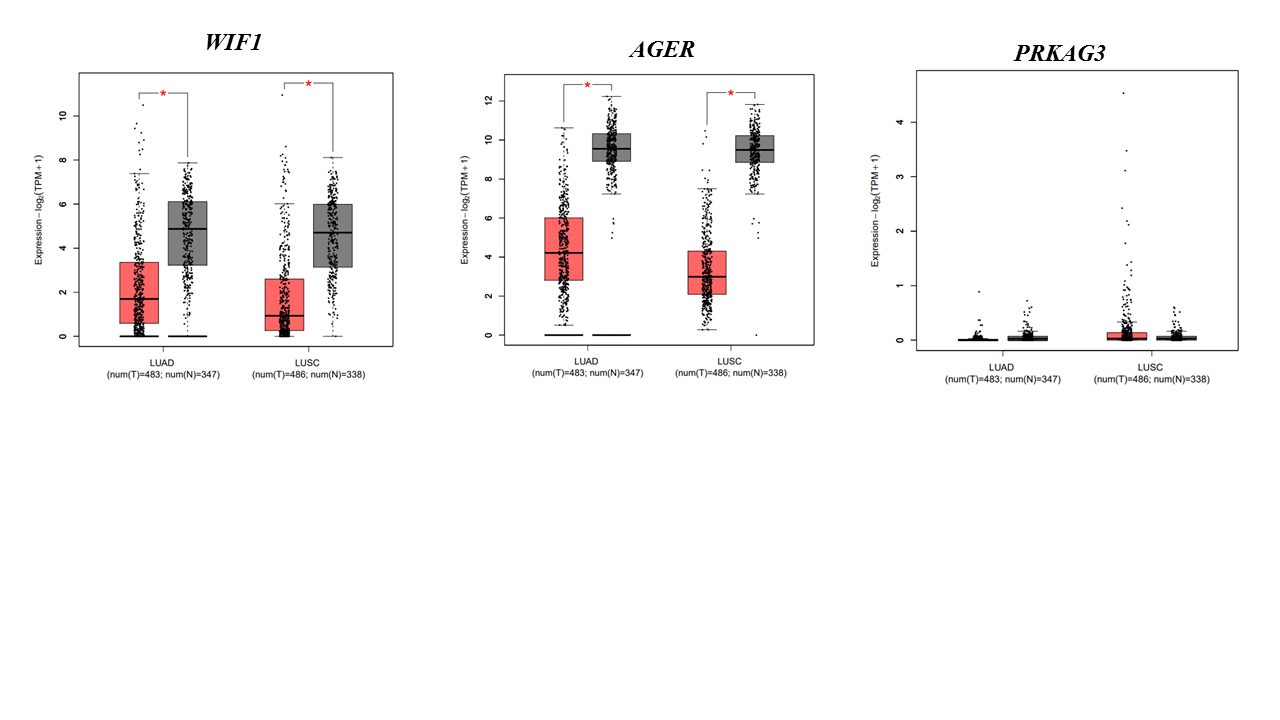


**Supplementary Figure 2:** GEPIA box plots show the expression patterns of nine significantly dysregulated genes (*OLR1, WNT10A, RASAL1, RAMP3, GADD45B, KLF4, WIF1, AGER,* and *PRKAG3*) common between sarcoidosis and lung cancer.


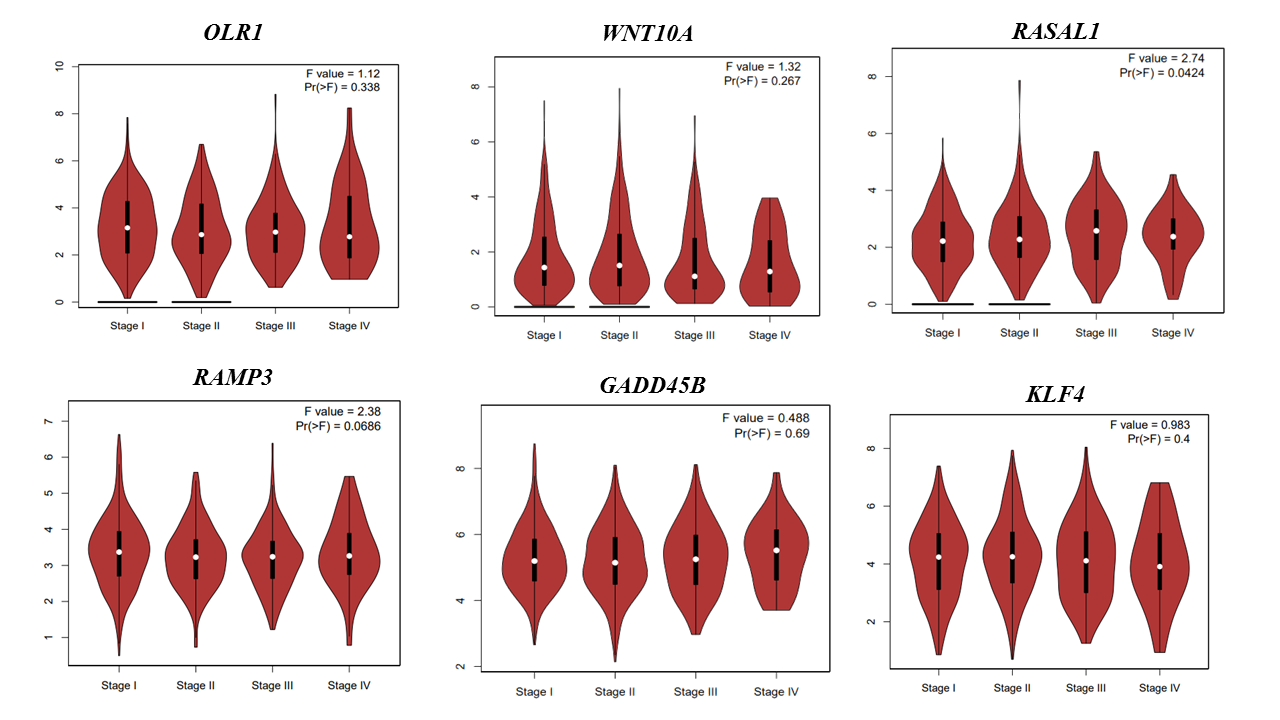


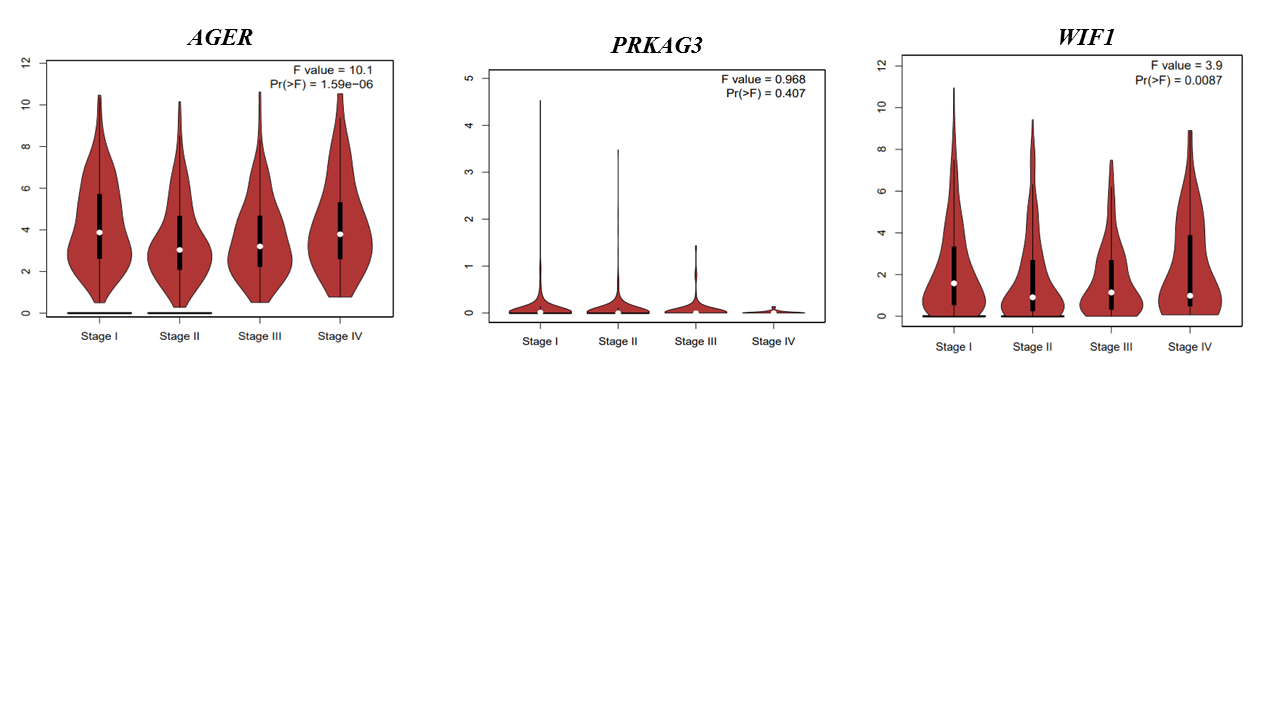


**Supplementary Figure 3:** GEPIA violin plots showing gene expression across pathological stages based on TCGA clinical annotations for common genes shared between sarcoidosis and lung cancer (*OLR1, WNT10A, RASAL1, RAMP3, GADD45B, KLF4, WIF1, AGER,* and *PRKAG3*).


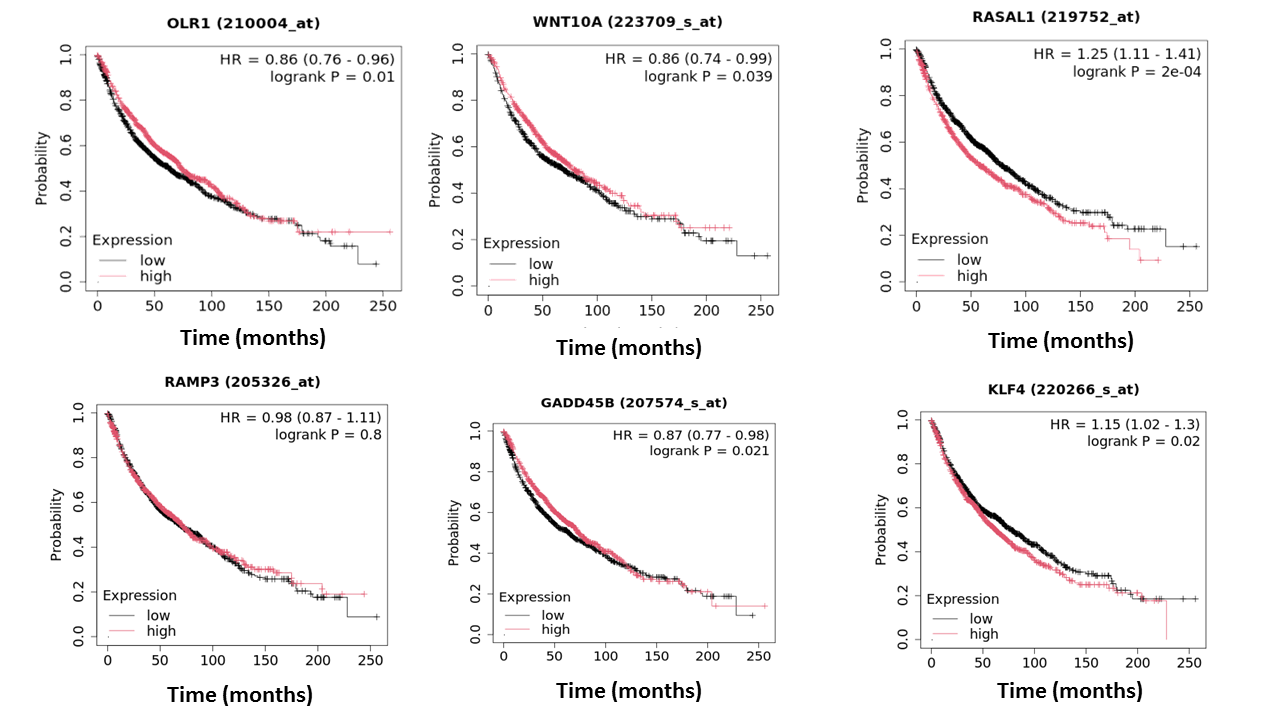


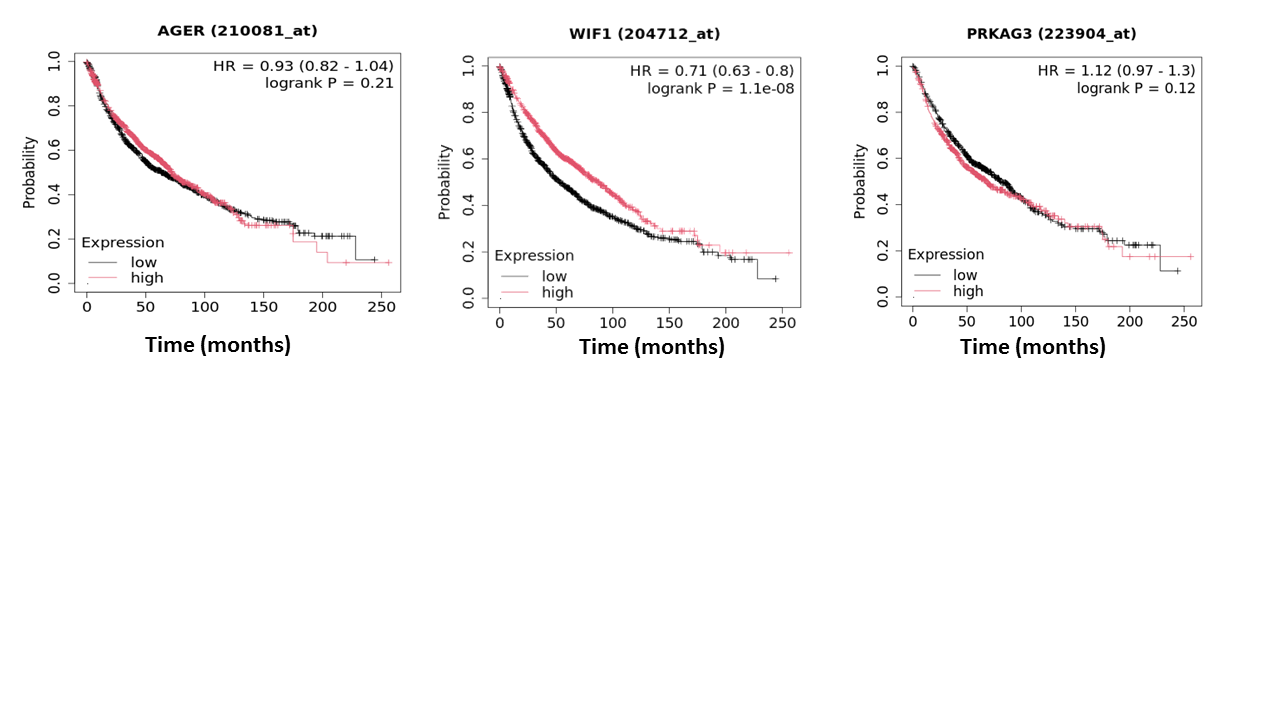


**Supplementary Figure 4:** Kaplan-Meier survival analysis revealed that higher expression levels of *WNT10A*, *RASAL1*, and *KLF4* were significantly associated with poor survival in lung cancer patients. In contrast, decreased expression of *OLR1*, *GADD45B*, and *WIF1* correlated with higher mortality


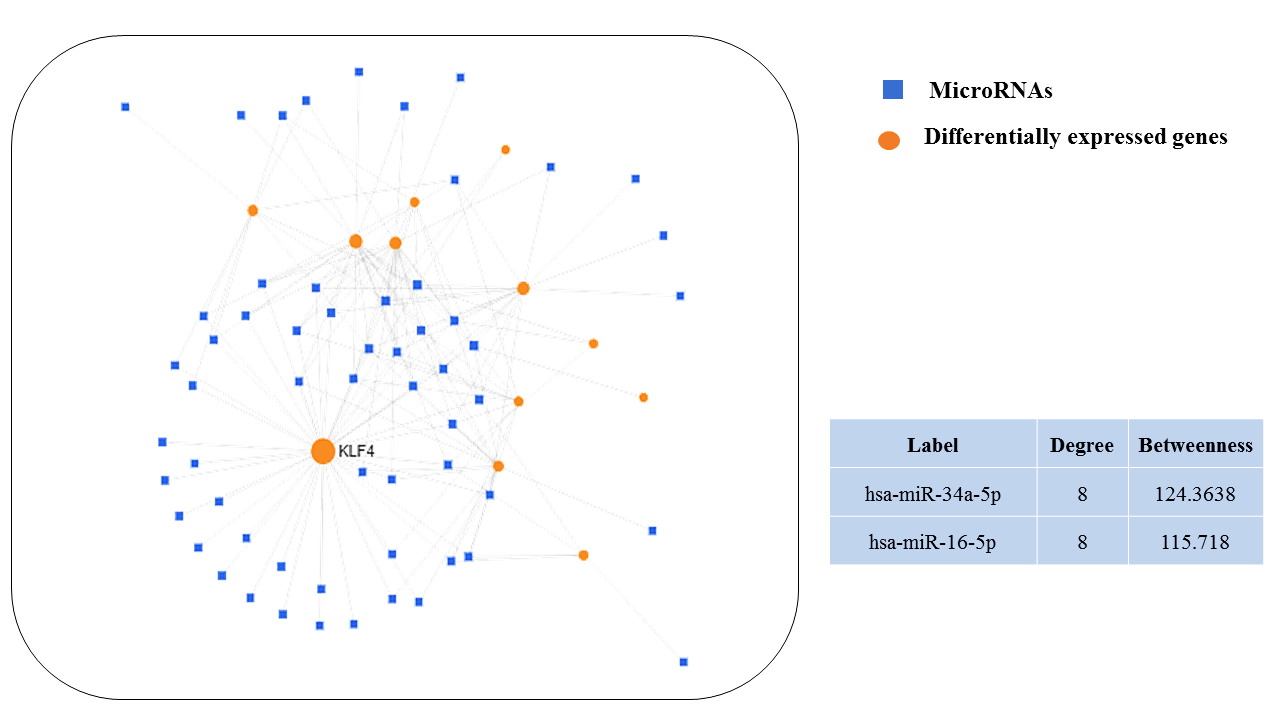


**Supplementary Figure 5:** Gene-miRNA network. Gene-miRNA network analysis revealed that hsa-mir-34a-5p and hsa-mir-16-5p exhibited the highest association with the common genes based on degree centrality (cut-off value = 8). The complete network consisted of 71 nodes and 167 edges.


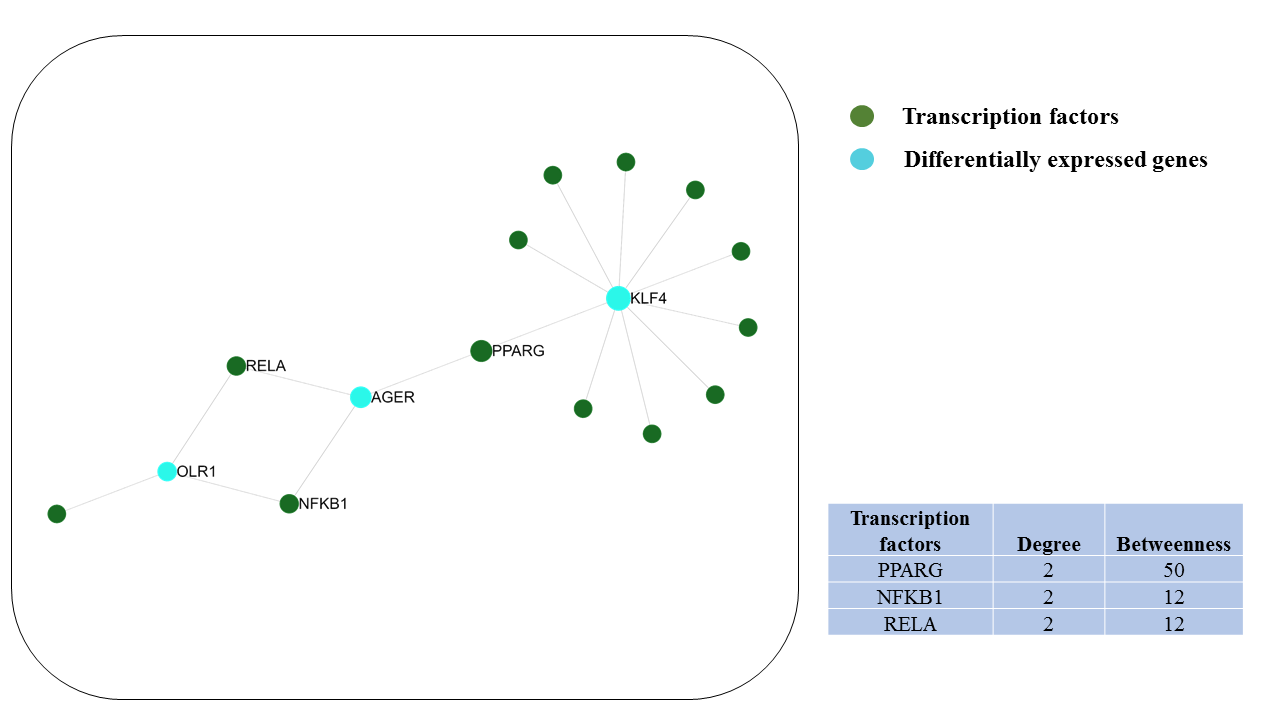


**Supplementary Figure 6:** Gene-transcription factor network analysis identified PPARG, NFKB1, and RELA as the top transcription factors interacting with the common differentially expressed genes (DEGs) between sarcoidosis and lung cancer, based on a degree centrality cut-off value of 2.
